# Sex-Specific Differences in Severity of Depressive Symptoms, Heart Rate Variability, and Neurocognitive Profiles of Depressed Young Adults: Exploring Characteristics for Mild Depression

**DOI:** 10.3389/fpsyt.2020.00217

**Published:** 2020-03-17

**Authors:** Jae-A Lim, Je-Yeon Yun, Yoobin Choi, Soo-Hee Choi, Yoonhee Kwon, Hwa Young Lee, Joon Hwan Jang

**Affiliations:** ^1^ Department of Psychiatry, Seoul National University Hospital Biomedical Research Institute, Seoul, South Korea; ^2^ Department of Psychiatry, Seoul National University Health Service Center, Seoul, South Korea; ^3^ Seoul National University Hospital, Seoul, South Korea; ^4^ Yeongeon Student Support Center, Seoul National University College of Medicine, Seoul, South Korea; ^5^ Department of Psychiatry, Seoul National University Hospital, Seoul, South Korea; ^6^ Department of Medicine, Seoul National University College of Medicine, Seoul, South Korea

**Keywords:** young adults, mild depressive symptom, heart rate variability, sex differences, emotion regulation

## Abstract

Mild depressive symptoms (MDS) reflect vulnerability to major depression that does not meet the criteria for a major depressive disorder (MDD). Previous research indicates that it is difficult to identify MDS in young adults, and they exhibit diverse aspects of depressive symptoms caused by clinical depression, which can lead to poor academic performance, relationship difficulties, and even suicide. Additionally, many young adults remain unaware of their depressive symptoms during the early stages of MDD. Thus, the present study investigated clinical, neurocognitive, and physiological characteristics of young adults with various symptoms of depression and explored sex-specific differences. A total of 113 students aged 18–35 (MDD: n = 32; MDS: n =37; control [CON]: n = 44) participated in the study. Self-report clinical measures, short-term cardiac activity measured by finger sensors, and neurocognitive data were collected. Pearson's correlations, two-way analysis of variance (ANOVA), principal component analysis, and exploratory structural equation modeling were conducted for the statistical analyses. Furthermore, the measurement invariance of the latent factor model was tested, and fit indices were compared according to sex. The results revealed that male students showed greater sympatho-vagal activity than female students. Additionally, male MDS students tended to exhibit decreased performance levels in neurocognitive function tasks compared with MDD and CON males, whereas female MDS students showed distinct characteristics compared to MDD and CON females on self-report measures of anxiety. Correlation analyses identified a positive association between the level of anger perception and latency in the executive function test among both males and females. Additionally, the use of a structured model revealed significant sex-specific differences in factor estimates. The present results suggest that recognizing the early signs of MDS that account for sex-specific differences in both subjective and objective measures may improve the diagnosis and monitoring of young adults with MDS.

## Introduction

College students frequently experience personal problems. Common stressors include the struggle to establish one's identity in a new environment, academic demands, and changes in social life (1). A recent review indicated that mental health problems often begin during this transition period (2). Approximately 20%–30% of college students who were not receiving psychiatric services reported a significant experience of depression at some time (3). Students with mental health problems report poorer relationships with other students and faculty members, lower levels of engagement in campus clubs and activities, lower grade averages, and lower graduation rates than students without mental health problems (4–8). In addition, depression during this period is more likely to be chronic, severe, disabling, and lead to suicide attempts than depression that develops in middle age (9). Although many students experience various degrees of depression, they often do not seek treatment. Many young adults are unaware of their symptoms of depression, especially during the early stages of the disease, but they may experience suicidal ideation later on (10). Major depressive disorder (MDD) has been studied extensively in the young adult population. However, identifying mild depressive symptoms (MDS) is crucial for implementing early interventions and improving prognoses.

Previous studies have reported that adolescent and young adult females are more prone to depression than their male counterparts (11, 12). Some comparisons showed that depressed girls were more likely to experience depression subtypes associated with anxiety, sleep/appetite disturbance, and feelings of failure, concentration problems, sadness/depressed mood, and health worries than depressed boys. Depressed boys were more likely to experience anhedonia and showed greater diurnal variations in mood and energy (9, 11). However, only a few studies have investigated sex-specific differences in the development of depression in adults in their 20s, and focusing on the impact of depression severity in young adults is considered essential for improving mental health care at universities. Both strongly positive and negative emotionality are significant predictors of adolescent depression (13). Depressed individuals show decreased initiation of and responsiveness to social contact, and a lack of interest in social interactions (14). In addition, various biological, psychological, genetic, and social explanations have been formulated to explain the higher rates of depression among women (15). Emotional processing studies in normal adolescents and adults showed that females were better than males at recognizing emotional expressions in videos that resembled real-life encounters (16). However, depression seems to have different effects on how females and males process emotions. Females with MDD processed nonverbal emotional cues (e.g., facial expressions) less accurately than did nondepressed females or both depressed and nondepressed males. In contrast, depressed males processed emotions equally well as nondepressed males (17).

Because university students rarely seek treatment for their depression symptoms, we must consider both subjective and objective assessment measures to formulate effective predictive markers for MDS. Heart rate variability (HRV) has emerged as a physiological marker for emotional regulation; however, it has rarely been used to investigate depression and anxiety in young adults (2, 18, 19). Furthermore, little is known about the early developmental trajectories of depression and general psychopathology in children and adolescents, and as a result, additional research that includes younger samples will be needed to explore these developmental pathways in greater detail (20). A meta-analysis of studies that compared resting-state HRV between unmedicated adults with major depression and controls suggested that patients with major depression are more likely to display small reductions in several measures of HRV, including high-frequency (HF) and low-frequency (LF) HRV and an increase in the LF/HF ratio (21). Furthermore, substantial cardiac autonomic control differences between the sexes have been reported in healthy subjects, with women exhibiting increased power in the HF band. This indicates that despite their higher mean heart rates, women show greater vagal activity than men (22, 23). Other studies have reported that symptoms of depression were more strongly associated with poor cardiac vagal control and sympathetic predominance among depressed males than females (24–26). Studies of sex-related differences in HRV among depressed young adults showed that depressed males had significantly lower HRV than healthy control (CON) males, whereas there were no significant differences in autonomic function between depressed females and CON females (24). Taken together, these previous findings provide evidence for sex-specific differences in HRV. Therefore, it is important to distinguish the unique HRV characteristics of young adult males and females with MDS/MDD. One technique that may enhance our understanding of the patterns associated with depression, including clinical parameters, social function, HRV, and neurocognitive parameters, is exploratory structural equation modeling (ESEM), which integrates the important advantages of exploratory factor analyses, confirmatory factor analysis, and structural equation model (27, 28). ESEM has wide applicability to all disciplines that are based on the measurement of latent constructs, a major advantage of ESEM is that it typically provides a better fit to the data (28). The ESEM approach represents how latent variables are related, so the specifications of a particular model should be estimated (29). Thus, assessments of goodness of fit and estimations of the parameters of the hypothesized model(s) are necessary (29).

The purpose of this study was to investigate overall sex-specific differences in MDS and MDD in early adulthood. In particular, we assessed the relationship between HRV, as an objective measurable marker, and clinical/neurocognitive variables. Based on previous findings, we hypothesized that both subjective and objective characteristics of depression would be separately influenced by group and sex, especially short-term cardiac activity and emotional regulation indices. In addition, we expected significant sex-specific differences between path coefficients from the proposed factor structure.

Previous studies investigating depression in university students have reported that problematic outcomes can result from increased levels of anxiety and decreased levels of social support and cognitive/academic functioning. If these clinical characteristics can be identified early in the development of depression, then the pathogenesis and progression of this disorder can be more clearly understood, with beneficial consequences for diagnosis and treatment. Therefore, the present study aimed to determine how the clinical characteristics of young adult MDD patients assessed in previous studies (e.g., depressed mood, anhedonia, severe recurrent verbal, or behavioral outbursts of temper three or more times per week) manifest in MDS populations. Furthermore, in addition to the current questionnaire, objective variables such as HRV and cognitive function were considered in an attempt to overcome the limitations of previous studies that depended on questionnaires.

## Methods

### Participants

In total, 113 undergraduate and graduate students including 45 males [mean age = 25.09; standard deviation (SD) = 2.98] and 68 females (mean age = 24.10; SD = 3.69) participated in this study. The students were encouraged to undergo regular health examinations, including mental health questionnaires such as the Patient Health Questionnaire-9 (PHQ-9), Generalized Anxiety Disorder-7 (GAD-7), and State-Trait Anxiety Inventory-State anxiety (STAI-S). Subsequently, potential MDS and MDD participants were sent messages *via* electronic mail encouraging them to see a mental health expert. Cross-sectional data were collected from May 2017 until July 2018 at Seoul National University, Seoul, South Korea.

Participants were eligible for the study if they were 18–35 years of age; had not used psychotropic medication within the 8 weeks prior to enrollment; had no history of psychosis, substance abuse or dependence; were able to provide written informed consent to participate; were not pregnant; and had no history of significant head injuries. The Mini-International Neuropsychiatric Interview (MINI) was administered to all participants, and in each case, psychiatrists (JHJ and JYY) confirmed the diagnosis through psychiatric interviews. Financial compensation was provided as a reward for participation.

Participants diagnosed with clinical depressive symptoms met at least one of the following criteria: PHQ-9 score ≥10 points; GAD-7 score ≥10 points; STAI-S score ≥61 points for males or ≥65 points for females (referred to the Korean validation study of STAI-S (30), we determined that it was to have a sex differences at the cut-off value); or a history of suicidal thought/attempt/plan within the past 6 months. In addition to meeting at least one depressive (PHQ-9) or anxiety (GAD-7/STAI-S) scale criterion, each MDD subject satisfied five or more category A criteria for MDD, and each MDS participant fulfilled one to four category A criteria for MDD. Next, the participants with clinical depressive symptoms were placed in either the MDD or MDS group. Additionally, each MDS and MDD participant answered “yes” to at least one of the following questions: “Have you been consistently depressed or down, most of the day, nearly every day, for the past 2 weeks?” and “In the past 2 weeks, have you been much less interested in most things or much less able to enjoy the things you used to enjoy most of the time?”. The MDD group consisted of 32 participants who met the *Diagnostic and Statistical Manual of Mental Disorders, Fifth Edition* (31) criteria for MDD. The remaining 37 participants were classified in the MDS group. Additionally, a total of 44 healthy CON participants with no Axis I psychiatric disorder (confirmed using the MINI) and who did not meet the criteria for clinical depressive symptoms described above were recruited using flyers.

This study was approved by the Institutional Review Board of Seoul National University College of Medicine and Hospital (Seoul, South Korea; No. 1608-079-785), and has therefore been performed in accordance with the ethical standards in the 1964 Declaration of Helsinki and its later amendments. All subjects provided written informed consent prior to participation.

### Study Design

The experimental procedures involved three parts:

1. A self-report questionnaire to assess the subject's clinical/psychological profile. The questionnaire consisted of two sections: the same mental health checkup tool (including PHQ-9, GAD-7 and STAI-S) and a questionnaire used for research. The mental health checkup questionnaire was waived if a participant had completed a health checkup within the previous month.
2. Resting-state HRV was monitored and recorded for 6 min with the participant sitting in a chair with the arm resting on a desk. The questionnaire and neurocognitive tests were conducted along with the HRV measurement; thus, coffee intake and cigarette use, which might affect HR measures, were not strictly prohibited before assessment. Additionally, because all participants were university students, the experiment was available during off-peak hours; thus, circadian rhythm was not controlled. During the HRV acquisition phase, participants were explicitly instructed to relax, move as little as possible, and refrain from meditating or thinking of something specific. A sensor was attached to the little finger of the nondominant hand, and the participant was asked to keep this arm as still as possible. Photoplethysmography (PPG) waveforms can easily be recorded from the finger and then digitized to compute reliable estimates of HRV (32). PPG-derived HRV data can provide a user-friendly self-monitoring system for MDD screening (33), and PPG-based methods can be used for short-term estimation of HRV as well as long-term monitoring of patients for diagnostic and prognostic purposes (32).
3. Computerized neurocognitive tests were performed to assess social cognition, attention, executive function, impulsivity, and working memory.

These tests were completed in approximately 2 h and 20 min. All participants were administered the tests at either one or two sessions according to each participant's schedule; all study tests were completed within 2 months.

## Measurements

### Questionnaires About Depression, Anxiety, and Clinical Characteristics

#### Patient Health Questionnaire-9 (PHQ-9)

The PHQ-9 (34, 35) is a nine-item instrument that screens for the presence and severity of depression. It asks the patient about their experiences over the preceding 2 weeks. Scores range from 0 to 27. In general, a score ≥10 suggests depression.

#### Center for Epidemiologic Studies Depression Scale (CES-D)

The CES-D (36, 37) is a 20-item instrument that asks the patient to rate how often they experienced symptoms associated with depression during the preceding week. Scores range from 0 to 60, with high scores indicating more severe symptoms.

#### State-Trait Anxiety Inventory-State Anxiety (STAI-S)

The STAI-S (30, 38) is a psychological inventory that measures state anxiety (i.e., anxiety about an event) and consists of 20 questions. Scores range from 20 to 80, and greater scores mean higher levels of anxiety.

#### Generalized Anxiety Disorder-7 (GAD-7)

GAD-7 (39, 40) is a self-report instrument to assess the severity of anxiety in general. GAD-7 has seven items and scores range from 0 to 21. Greater scores indicate greater anxiety over the preceding 2 weeks.

#### Resilience Appraisal Scale (RAS)

The RAS (41) is used to assess an individual's ability to cope with their emotions, solve problems, and acquire social support. It consists of 12 items, and scores range from 12 to 60. Greater scores indicate more positive self-appraisals.

#### Rosenberg Self Esteem Scale (RSES)

The RSES (42, 43) consists of 10 items that are answered using a four-point scale and measure feelings of worthiness. Scores range from 10 to 40, and greater scores indicate higher self-esteem.

#### Social Support Scale

The social support scale (44) is a 25-item questionnaire that measures perceptions of social support and satisfaction with interpersonal relationships. Scores range from 25 to 125, and greater scores indicate higher levels of social support.

#### World Health Organization Quality of Life (WHOQOL), Abbreviated Version

The WHOQOL (45, 46) instrument comprises 26 items that measure an individual's social relationships and their physical and psychological health in the context of their cultural environment. Greater scores indicate better quality of life.

#### Barratt Impulsiveness Scale (BIS)

The BIS (47, 48) is a questionnaire that assesses impulsiveness. It consists of 30 items, which assess attentional, motor, and nonplanning impulsiveness factors. Scores range from 30 to 120. Greater scores are associated with more impulsive behaviors and preferences.

#### Beck Hopelessness Scale (BHS)

The BHS (49, 50) is a 20-item inventory that measures three major aspects of hopelessness: feelings about the future, loss of motivation, and expectations. Scores range from 0 to 20, and greater scores indicate increased feelings of hopelessness.

#### Neuroticism-Extraversion-Openness (NEO) Five Factor Inventory

The NEO (51) is a personality inventory that examines a person's Big Five personality traits (openness to experience, conscientiousness, extraversion, agreeableness, and neuroticism). The shorter inventory, which we used in this study, scores 60 items using a 5-point scale (52, 53).

#### Pittsburgh Sleep Quality Index (PSQI)

The PSQI (54, 55) is a questionnaire that evaluates sleep quality during the preceding month. The PSQI consists of 18 questions, and scores range from 0 to 21. Greater mean scores indicate lower sleep quality, and scores >5 are associated with poor sleep quality.

#### Resting State HRV

HRV was measured using an MP150 System and AcqKnowledge software (ver. 5.0; BIOPAC Systems, Inc., Goleta, CA, USA). Prior to analysis, the amplitude heights of the acquired data were visually inspected to assess data quality. If a participant's data quality was found unsuitable for analysis, the HRV data were obtained again. Next, 6 min of resting-state data were processed in two steps. First, beats from the raw data were labeled using BIOPAC software, and R-R intervals containing rate information were extracted. Next, the R-R intervals were converted into American Standard Code for Information Interchange (ASCII) format, and the data were analyzed using Kubios HRV software ver. 3.0.2; (56). We also corrected for artifacts that were due to noise caused by movement or equipment malfunctions. If the results of the Kubios HRV output revealed that artifact correction was necessary, the artifacts were processed by selecting the “Threshold: custom” option embedded in the Kubios software. HRV analysis was performed in compliance with a standardized protocol.

#### Neurocognitive Function

Neuropsychological tests from the Cambridge Neuropsychological Test Automated Battery (CANTAB) were used to evaluate neurocognitive function (57). The tests included in this study were as follows:

1. The Emotion Recognition Task (ERT), which measures a subject's ability to identify six basic emotions from facial expressions.
2. The Rapid Visual information Processing (RVP) test, which provides a measure of sustained attention.
3. The One Touch Stockings of Cambridge (OTS) test, which assesses executive function. It assesses both the spatial planning and the working memory subdomains.
4. The Stop Signal Task (SST), which measures response inhibition (i.e., impulse control).
5. The Spatial Working Memory (SWM) test, which measures the retention and manipulation of visuospatial information.

### Data Analysis

All data analyses were performed using R software (ver. 3.5.1; R Development Core Team), Package ggplot2 was used for graphical representation, car and multcomp were used for the two-way analysis of variance (ANOVA) tests, Package Hmisc was used for calculating correlations, Package psych was used for the principal component analysis (PCA), and Package lavaan and semTools were used for the ESEM. A *P*-value <0.05 was considered to indicate statistical significance.

For each measurement, descriptive analyses were used to calculate the means and SDs for each group (CON vs. MDS vs. MDD), and the data were also analyzed according to sex (male vs. female). Differences between the mean scores for group × sex were calculated using two-way ANOVA.

For further analysis, all mean scores were Z-transformed into the range of 0 to 1 to include all data from the different sources on a single scale. To explain the results of the analysis more clearly and concisely, only variables that showed statistically significant group or sex differences in the two-way ANOVA were used in the subsequent analyses. The correlation between HRV and other clinical/neurocognitive variables were estimated according to sex using Pearson's correlation coefficient. Then, PCA was used to select variables from all the scales, and orthogonal (i.e., varimax) rotations were performed. A rotated factor loading value ≥0.50 was considered significant (58).

Next, ESEM was used to explore the structure of factors within the depression/anxiety and clinical/HRV/neurocognitive domains. Model fitness was evaluated using the following indicators: root mean-square error of approximation (RMSEA), comparative fit index (CFI), Tucker-Lewis index (TLI) and standard root mean square residual (SRMR) (29, 59).

In addition, measurement invariance tests between males and females were performed to confirm factor structure. These tests can identify differences among the factors and may be used to support the interpretation of fitted value differences between males and females.

## Results

### Clinical Variables, HRV, and Neurocognitive Characteristics According to Sex

A total of 45 males (39.82%) and 68 females (60.18%) were included in this study. No significant group × sex differences in age distribution were observed. The clinical scales, HRV, and the CANTAB test data are expressed as means ± SD in **Table 1**. The significant results from a 3 × 2 ANOVA separated by group (i.e., CON, MDS, and MDD) and sex (i.e., male and female) are shown in **Table 2**. Significant main and interaction effect plots are shown as supplementary data (**Figure S1**).

**Table 1:** Participants characteristics.

	Male (n=45)			Female (n=68)		
	CON (n=17)	MDS (n=15)	MDD (n=13)	CON (n=27)	MDS (n=22)	MDD (n=19)
	M ± SD	M ± SD	M ± SD	M ± SD	M ± SD	M ± SD
Age	24.7 ± 2.7	26.1 ± 3.2	24.5 ± 3.0	24.2 ± 3.8	23.9 ± 3.5	24.3 ± 4.0
**Questionnaires**						
PHQ-9	3.2 ± 2.6	6.7 ± 3.9	11.5 ± 4.1	3.7 ± 2.5	8.4 ± 3.6	9.6 ± 5.6
CES-D	11.5 ± 8.7	19.3 ± 7.8	33.3 ± 12.2	12.5 ± 7.9	23.9 ± 8.1	33.7 ± 9.9
STAI-S	44.1 ± 8.9	51.0 ± 8.9	57.6 ± 8.4	45.9 ± 9.1	55.3 ± 8.6	57.6 ± 8.0
GAD-7	2.9 ± 2.7	3.3 ± 2.6	9.4 ± 5.0	3.4 ± 2.8	7.7 ± 5.0	7.4 ± 4.1
RAS	43.2 ± 8.2	37.9 ± 5.4	33.8 ± 8.7	44.7 ± 7.2	36.5 ± 6.1	36.5 ± 9.0
RSES	30.3 ± 4.7	26.8 ± 5.0	25.8 ± 4.2	30.2 ± 5.5	24.9 ± 4.3	25.4 ± 4.6
Social support	97.2 ± 12.1	91.3 ± 13.0	78.0 ± 10.9	97.0 ± 13.8	86.1 ± 17.9	91.3 ± 8.4
WHOQOL total	82.8 ± 12.8	73.2 ± 13.5	61.1 ± 11.3	79.7 ± 12.7	69.1 ± 11.4	62.3 ± 9.8
BIS total	62.5 ± 10.9	65.5 ± 11.1	70.0 ± 12.5	60.8 ± 8.2	73.0 ± 10.7	70.1 ± 12.3
BHS	3.9 ± 4.4	7.1 ± 5.6	9.6 ± 6.3	4.4 ± 4.1	9.6 ± 5.3	9.3 ± 2.7
NEO agreeableness	42.1 ± 5.2	38.7 ± 4.5	33.2 ± 6.9	39.9 ± 4.5	37.4 ± 6.5	37.2 ± 4.8
NEO conscientiousness	37.7 ± 10.0	35.3 ± 6.8	32.6 ± 8.5	40.9 ± 5.9	35.7 ± 6.5	34.1 ± 8.9
NEO extraversion	40.4 ± 6.7	35.4 ± 7.1	32.7 ± 6.7	37.4 ± 5.8	33.8 ± 7.9	35.2 ± 8.6
NEO neuroticism	33.8 ± 9.9	38.9 ± 5.6	45.7 ± 4.9	38.4 ± 6.9	44.9 ± 8.5	47.3 ± 5.6
NEO openness	39.9 ± 5.7	42.0 ± 7.8	40.9 ± 7.1	42.8 ± 6.2	44.0 ± 7.3	42.1 ± 6.3
PSQI	6.2 ± 2.7	7.5 ± 2.5	10.0 ± 1.4	6.4 ± 1.9	8.6 ± 3.0	10.1 ± 3.8
**HRV** **(Time domain)**						
Mean RR (ms)	787.8 ± 116.1	829.4 ± 79.6	795.6 ± 133.7	832.0 ± 118.8	798.5 ± 106.7	807.3 ± 95.9
Mean HR (bpm)	77.8 ± 12.0	73.0 ± 7.0	77.4 ± 13.1	73.6 ± 11.0	76.4 ± 9.8	75.4 ± 9.2
RMSSD (ms)	44.6 ± 16.9	44.6 ± 14.4	40.4 ± 16.2	45.1 ± 16.5	44.4 ± 18.0	43.2 ± 18.7
**HRV** **(Frequency domain)**						
Power LF (ms^2^)	1,015.1 ± 915.2	1,045.5 ± 906.6	1,549.5 ± 1487.0	737.7 ± 1164.6	420.6 ± 274.7	488.3 ± 438.2
Power HF (ms^2^)	893.5 ± 803.8	718.4 ± 539.1	540.1 ± 422.5	726.4 ± 606.5	747.0 ± 853.8	807.3 ± 822.4
Total power (ms^2^)	1,985.0 ± 1639.8	1,841.3 ± 1058.0	2,192.4 ± 1665.7	1,534.0 ± 1571.2	1,230.6 ± 960.6	1,356.6 ± 1197.9
Power LF (%)	51.5 ± 16.4	53.2 ± 21.3	64.7 ± 16.4	39.7 ± 18.6	40.1 ± 17.4	38.1 ± 14.7
Power HF (%)	43.4 ± 17.5	40.8 ± 21.0	30.4 ± 14.4	55.8 ± 19.5	54.1 ± 19.8	56.4 ± 17.1
LF/HF ratio	1.5 ± 1.0	2.6 ± 3.4	3.7 ± 4.1	1.1 ± 1.4	1.0 ± 0.9	0.8 ± 0.6
**Neurocognitive test**						
ERTUHRH	0.6 ± 0.1	0.6 ± 0.2	0.7 ± 0.2	0.7 ± 0.1	0.7 ± 0.2	0.6 ± 0.1
ERTUHRS	0.5 ± 0.1	0.5 ± 0.2	0.5 ± 0.2	0.5 ± 0.1	0.5 ± 0.1	0.5 ± 0.1
ERTUHRF	0.1 ± 0.1	0.1 ± 0.1	0.2 ± 0.2	0.1 ± 0.1	0.1 ± 0.1	0.2 ± 0.1
ERTUHRA	0.3 ± 0.2	0.4 ± 0.2	0.4 ± 0.2	0.4 ± 0.2	0.3 ± 0.2	0.3 ± 0.1
ERTUHRSU	0.4 ± 0.1	0.5 ± 0.1	0.5 ± 0.1	0.5 ± 0.1	0.5 ± 0.1	0.5 ± 0.1
ERTUHRD	0.4 ± 0.2	0.4 ± 0.2	0.4 ± 0.2	0.5 ± 0.1	0.4 ± 0.2	0.4 ± 0.1
RVPA	0.97 ± 0.02	0.95 ± 0.02	0.96 ± 0.02	0.96 ± 0.02	0.97 ± 0.03	0.94 ± 0.03
RVPTM	5.7 ± 4.1	9.6 ± 5.4	7.7 ± 5.1	8.0 ± 5.2	7.2 ± 5.9	11.8 ± 6.9
OTSMCC4	1.1 ± 0.2	1.2 ± 0.3	1.1 ± 0.2	1.2 ± 0.3	1.3 ± 0.4	1.3 ± 0.5
OTSMLC4	8,904.8 ± 5,160.4	19,526.7 ± 17,497.9	12,080.0 ± 6,209.4	12,121.4 ± 6,144.2	12,237.9 ± 6,806.9	13,791.8 ± 12,919.5
SSTSSRT	187.1 ± 23.9	213.4 ± 37.3	197.9 ± 31.5	195.9 ± 31.6	204.0 ± 27.2	206.5 ± 42.1
SWMBE	8.1 ± 8.9	9.1 ± 11.2	8.5 ± 9.1	16.0 ± 13.2	15.8 ± 14.8	14.6 ± 12.7
SWMTE	8.1 ± 8.9	9.3 ± 11.5	9.2 ± 10.0	16.6 ± 13.5	16.1 ± 15.3	15.0 ± 13.0
SWMS	2.7 ± 2.2	3.4 ± 2.8	2.9 ± 2.5	5.0 ± 2.7	4.2 ± 2.8	5.0 ± 3.2

CON, Control; MDS, Mild Depressive Symptoms; MDD, Major Depressive Disorder; M, Mean; SD, Standard Deviation; PHQ-9, Patient Health Questionnaire-9; CES-D, Center for Epidemiologic Studies Depression Scale; STAI-S, State-Trait Anxiety Inventory-State anxiety; GAD-7, Generalized Anxiety Disorder-7; RAS, Resilience Appraisal Scale; RSES, Rosenberg Self Esteem Scale; WHOQOL, World Health Organization Quality of Life abbreviated version; BIS, Barratt Impulsiveness Scale; BHS, Beck Hopelessness Scale; NEO, Neuroticism-Extraversion-Openness; PSQI, Pittsburgh Sleep Quality Index; HRV, heart rate variability; RR, time interval between successive electrocardiogram R-waves; HR, Heart Rate; RMSSD, Root Mean Square of Successive RR interval Differences; LF, Low Frequency; HF, High Frequency; ERTUHRH, Emotion Recognition Task Unbiased Hit Rate Happiness; ERTUHRS, Emotion Recognition Task Unbiased Hit Rate Sadness; ERTUHRF, Emotion Recognition Task Unbiased Hit Rate Fear; ERTUHRA, Emotion Recognition Task Unbiased Hit Rate Anger; ERTUHRSU, Emotion Recognition Task Unbiased Hit Rate Surprise; ERTUHRD, Emotion Recognition Task Unbiased Hit Rate Disgust; RVPA, Rapid Visual information Processing A prime; RVPTM, Rapid Visual information Processing Total Misses; OTSMCC4, One Touch Stockings of Cambridge Mean Choices to Correct (4 move); OTSMLC4, One Touch Stockings of Cambridge Mean Latency to Correct (4 move); SSTSSRT, Stop Signal Task Stop Signal Reaction Time; SWMBE, Spatial Working Memory Between Errors; SWMTE, Spatial Working Memory Total Errors; SWMS, Spatial Working Memory Strategy.

**Table 2:** Significant results for two-way analysis of variance.

Variable	Variance	SS	*df*	*F*	*P*	Pairwise comparisons
Questionnaires						
PHQ-9	Group	507.98	2	17.936***	<.001	MDD > MDS > CON
	Sex	1.94	1	0.137	0.712	
	Group × Sex	55.93	2	1.975	0.144	
CES-D	Group	3540.6	2	21.785***	<.001	MDD > MDS > CON
	Sex	11.1	1	0.137	0.712	
	Group × Sex	86.7	2	0.533	0.588	
STAI-S	Group	1353	2	9.003***	<.001	MDD=MDS > CON
	Sex	31	1	0.418	0.520	
	Group × Sex	79	2	0.524	0.594	
GAD-7	Group	366.69	2	12.720***	<.001	MDD > MDS > CON
	Sex	2.27	1	0.157	0.692	
	Group × Sex	179.85	2	6.239**	0.003	MDD : Male > CON : Male, MDS : Female > CON : Male, MDD : Female > CON : Male, MDD : Male > MDS : Male, MDS : Female > MDS : Male, MDD : Female > MDS : Male, MDD : Male > CON : Female, MDS : Female > CON : Female, MDD : Female > CON : Female
RAS	Group	667	2	5.975**	0.003	CON > MDS=MDD
	Sex	26	1	0.457	0.500	
	Group × Sex	77	2	0.687	0.505	
RSES	Group	174.3	2	3.775*	0.026	CON > MDS=MDD
	Sex	0.1	1	0.005	0.942	
	Group × Sex	17.1	2	0.371	0.691	
Social support	Group	2767	2	7.758***	<.001	CON > MDS=MDD
	Sex	0	1	0.002	0.966	
	Group × Sex	1482	2	4.156*	0.018	CON : Male > MDD : Male, CON : Female > MDD : Male
WHOQOL total	Group	3465	2	12.068***	<.001	CON > MDS > MDD
	Sex	97	1	0.673	0.414	
	Group × Sex	127	2	0.442	0.644	
BHS	Group	242.56	2	5.453**	0.006	MDD=MDS > CON
	Sex	1.76	1	0.079	0.779	
	Group × Sex	36.04	2	0.810	0.447	
NEO agreeableness	Group	586.3	2	10.129***	<.001	CON > MDD
	Sex	50.8	1	1.755	0.188	
	Group × Sex	190.9	2	3.297*	0.041	CON : Male > MDD : Male, CON : Female > MDD : Male
NEO extraversion	Group	466.8	2	4.574*	0.012	CON > MDS=MDD
	Sex	91.9	1	1.800	0.183	
	Group × Sex	138.9	2	1.361	0.261	
NEO neuroticism	Group	1048.1	2	9.976***	<.001	MDD > MDS > CON
	Sex	228.5	1	4.349*	0.039	Female > Male
	Group × Sex	84.5	2	0.805	0.450	
PSQI	Group	106.01	2	7.397***	<.001	MDD > MDS > CON
	Sex	0.46	1	0.064	0.801	
	Group × Sex	5.57	2	0.389	0.679	
**HRV** **(Frequency domain)**						
LF/HF ratio	Group	27.64	2	3.159*	0.047	NS
	Sex	1.38	1	0.315	0.576	Male > Female
	Group × Sex	22.4	2	2.560	0.083	MDD : Male > CON : Female, MDD : Male > MDS : Female, MDD : Male > MDD : Female
**Neurocognitive test**						
ERTUHRA	Group	0.12804	2	2.168	0.120	NS
	Sex	0.11893	1	4.027*	0.047	NS
	Group × Sex	0.18415	2	3.118*	0.048	NS
RVPTM	Group	121.1	2	1.979	0.143	
	Sex	53.1	1	1.737	0.190	
	Group × Sex	190.2	2	3.108*	0.049	MDD : Female > CON : Male
OTSMLC4	Group	9.29E+08	2	4.905**	0.009	NS
	Sex	1.08E+08	1	1.140	0.288	NS
	Group × Sex	5.92E+08	2	3.126*	0.048	MDS : Male > CON : Male
SWMBE	Group	9.2	2	0.031	0.970	
	Sex	664	1	4.442*	0.037	Female > Male
	Group × Sex	17.5	2	0.059	0.943	
SWMTE	Group	13.6	2	0.043	0.958	
	Sex	742.7	1	4.676*	0.033	Female > Male
	Group × Sex	33	2	0.104	0.902	
SWMS	Group	4.57	2	0.311	0.733	
	Sex	55.95	1	7.615**	0.007	Female > Male
	Group × Sex	11.51	2	0.784	0.459	

Tukey's method is used for multiple comparisons with all possible pairwise differences of means.

SS, Sum of Squares; df, degrees of freedom; CON, Control; MDS, Mild Depressive Symptoms; MDD, Major Depressive Disorder; PHQ-9, Patient Health Questionnaire-9; CES-D, Center for Epidemiologic Studies Depression Scale; STAI-S, State-Trait Anxiety Inventory-State anxiety; GAD-7, Generalized Anxiety Disorder-7; RAS, Resilience Appraisal Scale; RSES, Rosenberg Self Esteem Scale; WHOQOL, World Health Organization Quality of Life abbreviated version; BHS, Beck Hopelessness Scale; NEO, Neuroticism-Extraversion-Openness; PSQI, Pittsburgh Sleep Quality Index; HRV, heart rate variability; LF, Low Frequency; HF, High Frequency; ERTUHRA, Emotion Recognition Task Unbiased Hit Rate Anger; RVPTM, Rapid Visual information Processing Total Misses; OTSMLC4, One Touch Stockings of Cambridge Mean Latency to Correct (4 move); SWMBE, Spatial Working Memory Between Errors; SWMTE, Spatial Working Memory Total Errors; SWMS, Spatial Working Memory Strategy; NS, Not Significant.

*P < 0.05; **P < 0.01; ***P < 0.001.

For the clinical scales, significant main and interaction effects were found for the GAD-7 scale [for group: *F* (2, 107) = 12.720, *P* < 0.001; for interaction: *F* (2, 107) = 6.239, *P* = 0.003], Social Support scale [for group: *F* (2, 107) = 7.758, *P* < 0.001; for interaction: *F* (2, 107) = 4.156, *P* = 0.018], and the NEO agreeableness scale [for group: *F* (2, 107) = 10.129, *P* < 0.001; for interaction: *F* (2, 107) = 3.297, *P* = 0.041].

The results of the GAD-7 scale revealed that anxiety levels increased as depression became more severe. Scores for the MDD group were significantly higher than those for the MDS (*P* = 0.039) and CON (*P* < 0.001) groups, and the MDS students scored higher than the CON students (*P* = 0.005). Additionally, male MDD students scored significantly higher than male CON (*P* < 0.001), male MDS (*P* < 0.001), and female CON (*P* < 0.001) students. Furthermore, the female MDD group scored significantly higher than the male CON (*P* = 0.008), male MDS (*P* = 0.026), and female CON (*P* = 0.009) groups, and the female MDS group scored higher than the male CON (*P* = 0.002), male MDS (*P* = 0.008), and female CON (*P* = 0.001) groups.

For the Social support scores, the CON groups scored significantly higher than the MDD (*P =* 0.001) and MDS (*P =* 0.010) groups. Additionally, there were significant interactions between the male CON and male MDD (*P =* 0.002) groups and between the female CON and male MDD (*P* < 0.001) groups. Both the male and female CON groups had higher mean scores than the male MDD group.

The NEO agreeableness scores showed significant decreases in the MDD groups compared to the CON groups (*P* < 0.001). For the interaction, the male MDD groups had significantly lower scores than the male CON (*P* < 0.001) and female CON (*P =* 0.004) groups.

There were also significant main effects for the groups were: PHQ-9 [*F* (2, 107) = 17.936, *P* < 0.001], CES-D, [*F* (2, 102) = 21.785, *P* < 0.001], STAI-S [*F* (2, 107) = 9.003, *P* < 0.001], RAS [*F* (2, 107) = 5.975, *P* = 0.003], RSES [*F* (2, 107) = 3.775, *P* = 0.026], WHOQOL [*F* (2, 107) = 12.068, *P* < 0.001], BHS [*F* (2, 107) = 5.453, *P* = 0.006], NEO extraversion [*F* (2, 107) = 4.574, *P* = 0.012], and PSQI [*F* (2, 106) = 7.397, *P* < 0.001] scores. The depression, anxiety, and hopelessness scores increased significantly as depression became more severe. In contrast, average scores for resilience, self-esteem, quality of life, and extraversion decreased as depression became more severe.

In addition, NEO neuroticism scale showed main effects for both group and sex [for group: *F* (2, 107) = 9.976, *P* < 0.001; for sex: *F* (1, 107) = 4.349, *P* = 0.039]. Post-hoc analyses revealed that the MDD group had higher levels of neuroticism than the MDS (*P =* 0.047) and CON (*P* < 0.001) groups and that the MDS students scored higher than CON students (*P* = 0.001). Female students had higher scores than male students (*P =* 0.003).

For HRV, there was a significant main effect for group in the LF/HF ratio [*F* (2, 96) = 3.159, *P* = 0.047], but no differences for the between-group comparisons. A simple main effects analysis showed that the LF/HF ratio was greater for male than for female students (*P* < 0.001). In addition, although the interaction effects were not statistically significant, the mean value for the male MDD group was greater than those for the female MDD (*P =* 0.005), female MDS (*P =* 0.011), and female CON (*P =* 0.013) groups.

For the neurocognitive data, there was a significant interaction effect on RVP total misses (TM) [*F* (2, 107) = 3.108, *P* = 0.049], with the female MDD group making more errors than the male CON group in the attention tasks (*P =* 0.016).

There were significant main and interaction effects for the variables ERT unbiased hit rate anger [UHRA; for sex: *F* (1, 106) = 4.027, *P* = 0.047; for interaction: *F* (2, 106) = 3.118, *P* = 0.048] and the OTS mean latency to correct response [MLC4; for group: *F* (2, 107) = 4.905, *P* = 0.009; for interaction: *F* (2, 107) = 3.126, *P* = 0.048]. For the ERT UHRA, there were no significant differences in the pairwise comparisons. For the OTSMLC4 variable, there was a significant interaction between the male MDS and male CON groups (*P =* 0.030), with the mean latency of the male MDS group being slower than that of the male CON group.

Finally, significant main effects of sex were observed for SWM between errors (BE) [*F* (1, 107) = 4.442, *P* = 0.037], SWM total errors (TE) [*F* (1, 107) = 4.676, *P* = 0.033], and SWM strategy (S) [*F* (1, 107) = 7.615, *P* = 0.007]. Male students performed better on SWM tasks than female students.

### Correlations Between HRV, Clinical Measure, and Neurocognitive Data

Female students exhibited positive correlations of the ERT UHRA with scores on the RAS, RSES, and WHOQOL; there were no significant correlations in males. In both male and female students, there was a significant positive correlation between the ERT UHRA and the OTS MLC4. The only HRV measure included in the correlation analyses was the LF/HF ratio, which showed no significant correlations with any measure.

**Figure 1:**
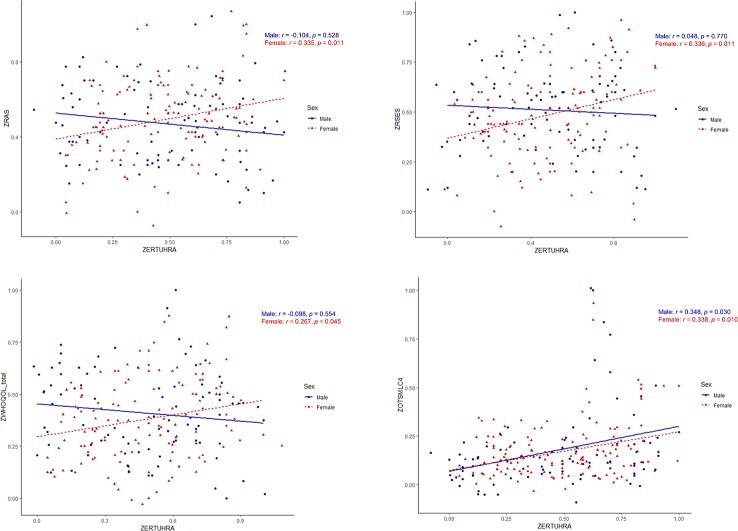
Correlation plots between neurocognitive task measuring anger perception, clinical characteristics, and neurocognitive task assessing executive function. Data derived from males are shown as blue straight lines, whereas data corresponding to females are shown as red dotted lines. ERTUHRA, Emotion Recognition Task Unbiased Hit Rate Anger; RAS, Resilience Appraisal Scale; RSES, Rosenberg Self Esteem Scale; WHOQOL, World Health Organization Quality of Life abbreviated version; OTSMLC4, One Touch Stockings of Cambridge Mean Latency to Correct (4 move).

The significant correlation plots are shown in **Figure 1**, and the characteristics of the other variables are shown in the supplementary data (**Table S1**).

### Principal Components and Relationships Among Latent Factors According to Sex

Initially, we used PCA to determine the factor structure among the different measures. We determined the number of principal components using the proportion of variance. Three factors were identified and varimax rotation provided factor loading that corresponded to the principal components. Items were allocated to each factor as shown in the supplementary data (**Table S2**). Self-report clinical measures loaded on the PC1 and PC3 factor, and HRV indexes did not load on any of the factors. Neurocognitive measures related to SWM were loaded onto the PC2 factor.

**Figure 2:**
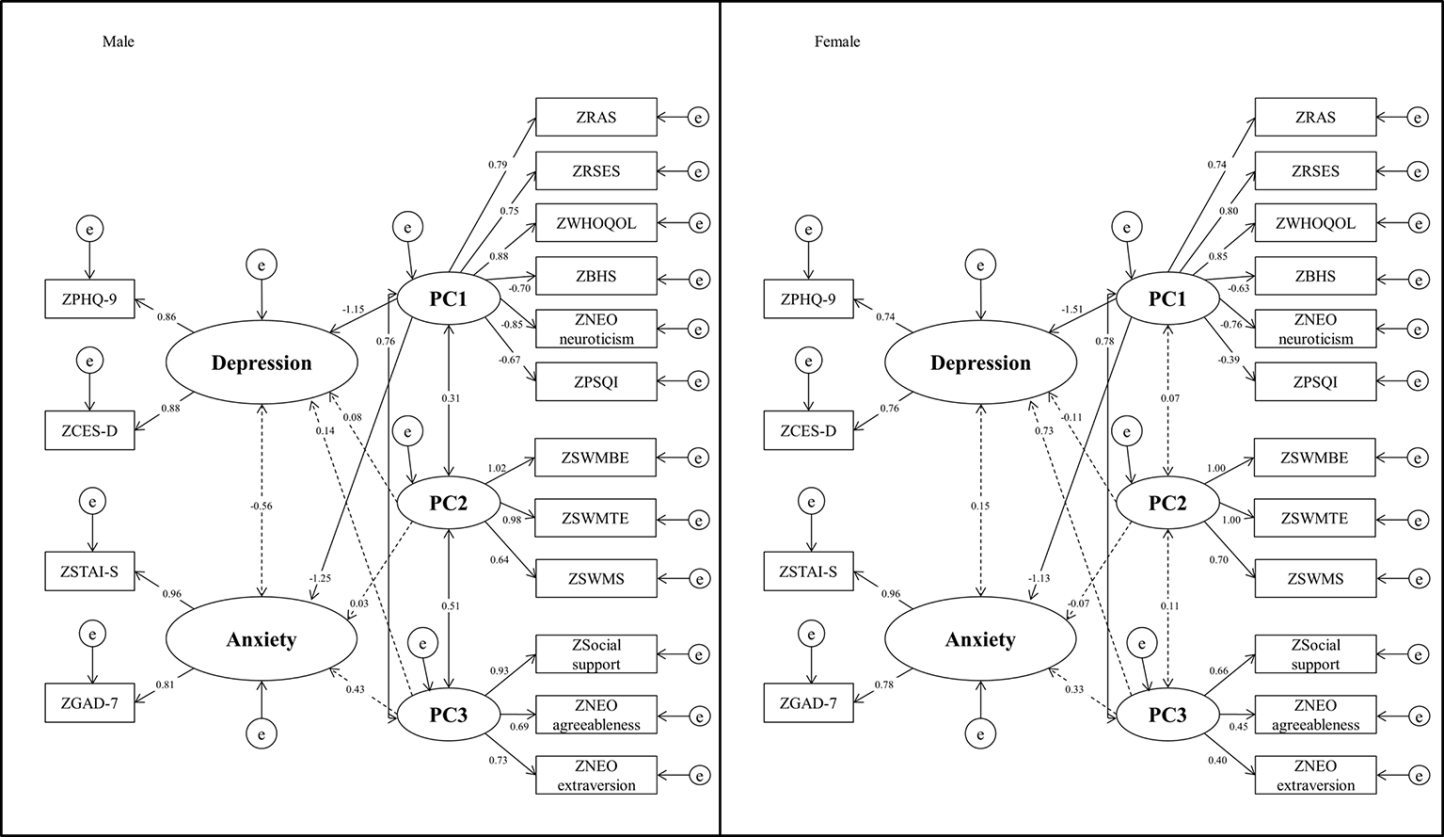
Exploratory structural equation model for clinical/HRV/neurocognitive variables across sex. All scales were Z-transformed. Standardized loadings are reported. For item loading, see **Table 3**. e, error.

We used ESEM as part of a subsequent approach to test the three-factor structured model. We used the PCA results to generate the structured model and fitted the same model for male and female students. The path diagram is shown graphically in **Figure 2**, and **Table 3** shows factor loading for measures in the latent dimension and substantial inter-factor correlations. In addition, we tested the model for goodness-of-fit. If the data were continuous, values of RMSEA <0.06, CFI >0.95, TLI >0.95, and SRMR <0.08 indicated an acceptable fit (29, 59). The present results were as follows: RMSEA = 0.101, CFI = 0.932, TLI = 0.913, and SRMR = 0.056. Thus, these results were marginal in terms of meeting the “acceptable” cutoff criterion.

**Table 3:** Three factor exploratory structural equation model with standardized factor loadings, comparison of latent means by sex.

	Estimate (Male)	SE	*P*	Estimate (Female)	SE	*P*
**Latent Variables**						
**Depression**						
ZPHQ-9	0.86			0.74		
ZCES-D	0.88	0.156	< 0.001	0.76	0.198	< 0.001
**Anxiety**						
ZSTAI-S	0.96			0.96		
ZGAD-7	0.81	0.139	< 0.001	0.78	0.117	< 0.001
**PC1**						
ZRAS	0.79			0.74		
ZRSES	0.75	0.163	< 0.001	0.80	0.166	< 0.001
ZWHOQOL total	0.88	0.163	< 0.001	0.85	0.146	< 0.001
ZBHS	−0.70	0.24	< 0.001	−0.63	0.193	< 0.001
ZNEO neuroticism	−0.85	0.158	< 0.001	−0.76	0.148	< 0.001
ZPSQI	−0.67	0.154	< 0.001	−0.39	0.174	0.002
**PC2**						
ZSWMBE	1.02			1.00		
ZSWMTE	0.98	0.022	< 0.001	1.00	0.013	< 0.001
ZSWMS	0.64	0.157	< 0.001	0.70	0.099	< 0.001
**PC3**						
ZSocial support	0.93			0.66		
ZNEO agreeableness	0.69	0.170	< 0.001	0.45	0.238	0.005
ZNEO extraversion	0.73	0.149	< 0.001	0.40	0.276	0.020
**Regressions**						
PC1à Depression	−1.15	0.248	< 0.001	−1.51	0.46	0.002
PC2à Depression	0.08	0.076	0.304	−0.11	0.075	0.407
PC3à Depression	0.14	0.207	0.480	0.73	0.646	0.181
PC1à Anxiety	−1.25	0.259	< 0.001	−1.13	0.361	< 0.001
PC2à Anxiety	0.03	0.098	0.766	−0.07	0.065	0.470
PC3à Anxiety	0.43	0.257	0.070	0.33	0.509	0.354
**Covariances**						
Depression <–> Anxiety	−0.56	0.002	0.553	0.15	0.003	0.855
PC1 <–> PC2	0.31	0.004	0.041	0.07	0.005	0.594
PC1 <–> PC3	0.76	0.006	0.001	0.78	0.004	0.001
PC2 <–> PC3	0.51	0.005	0.002	0.11	0.005	0.519
**Variances**						
ZPHQ-9	0.26	0.003	0.001	0.46	0.004	< 0.001
ZCES-D	0.22	0.004	0.002	0.42	0.005	< 0.001
ZSTAI-S	0.09	0.003	0.270	0.08	0.002	0.248
ZGAD-7	0.35	0.004	< 0.001	0.40	0.004	< 0.001
ZRAS	0.38	0.004	< 0.001	0.45	0.004	< 0.001
ZRSES	0.44	0.004	< 0.001	0.36	0.003	< 0.001
ZWHOQOL total	0.22	0.002	< 0.001	0.28	0.002	< 0.001
ZBHS	0.51	0.009	< 0.001	0.60	0.006	< 0.001
ZNEO neuroticism	0.27	0.003	< 0.001	0.42	0.003	< 0.001
ZPSQI	0.55	0.004	< 0.001	0.85	0.007	< 0.001
ZSWMBE	−0.04	0.001	0.019	0.01	0.001	0.278
ZSWMTE	0.05	0.001	0.006	−0.01	0.001	0.624
ZSWMS	0.59	0.007	< 0.001	0.52	0.007	< 0.001
ZSocial support	0.14	0.003	0.095	0.56	0.005	0.001
ZNEO agreeableness	0.52	0.006	< 0.001	0.80	0.005	< 0.001
ZNEO extraversion	0.46	0.004	< 0.001	0.84	0.006	< 0.001

SE, Standard Error; PHQ-9, Patient Health Questionnaire-9; CES-D, Center for Epidemiologic Studies Depression Scale; STAI-S, State-Trait Anxiety Inventory-State anxiety; GAD-7, Generalized Anxiety Disorder-7; PC, Principal Component; RAS, Resilience Appraisal Scale; RSES, Rosenberg Self Esteem Scale; WHOQOL, World Health Organization Quality of Life abbreviated version; BHS, Beck Hopelessness Scale; NEO, Neuroticism-Extraversion-Openness; PSQI, Pittsburgh Sleep Quality Index; SWMBE, Spatial Working Memory Between Errors; SWMTE, Spatial Working Memory Total Errors; SWMS, Spatial Working Memory Strategy.

To investigate the latent relationships among depression, anxiety, and the principal components, latent variables associated with depression and anxiety were constructed. The PHQ-9 and CES-D scales were used as depression factors, and the STAI-S and GAD-7 scales were used as anxiety factors.

All of the loaded variables for each of the three latent variables were large and statistically significant. In males, PC1 significantly predicted depression (standardized beta = –1.15) and anxiety (standardized beta = –1.25) latent variables, and for female students, PC1 significantly predicted depression (standardized beta = –1.51) and anxiety (standardized beta = –1.13).

Next, the same latent model was compared across sexes using Chi-squared difference tests. The difference in fit of the measures is shown in **Table 4**; there was a significant difference in the fit means between males and females.

**Table 4:** Comparison of proposed exploratory structural equation model in factor estimations according to sex.

Comparison models	χ^2^	*df*	AIC	BIC	CFI	*P* (>χ^2^)
Model 1 (fit. loadings)	362.38	199	−1802.9	−1522.3	0.897	0.663
Model 2 (fit. intercepts)	377.68	210	−1809.6	−1558.4	0.894	0.169
Model 3 (fit. means)	391.89*	215	−1805.4	−1567.5	0.888	0.014

χ^2^, Chi-square; df, degrees of freedom; AIC, Akaike Information Criterion; BIC, Bayesian Information Criterion; CFI, Comparative Fit Index.

*P < 0.05.

## Discussion

This study integrated both experimental and theoretical approaches to sex-specific predictive markers for depression. We compared MDS with MDD in university students and made the following observations:

1. The results of a two-way ANOVA indicated that male students had greater LF/HF ratios than female students. On the other hand, female students showed increased neuroticism scores compared to male students. Although the differences were not statistically significant, the male MDS group had the greatest mean latency on the OTS task and also exhibited decreased response inhibition and SWM relative to the male MDD and CON groups. Furthermore, the female MDS students scored highest on the GAD-7, BIS, and BHS and lowest in terms of resilience, social support, and extraversion compared to the female MDD and CON groups.
2. A correlation analysis revealed that, in female students, the level of anger perception was positively associated with the resilience, self-esteem, and quality of life scores. Additionally, both males and females showed positive relationships between levels of anger perception and latency in the executive function task.
3. The postulated latent factors (i.e., depression, anxiety, and the three principal factors) showed significant degrees of factorial invariance across the sexes.

### Implications of the Two-Way ANOVA Results

Previous research (22, 24, 25) reported a stronger association between symptoms of depression and poor cardiac vagal control in males than in females. These results are consistent with findings that suggest males and females use different strategies to cope with everyday stress and that this may result in different HRV in each sex (22). A high LF/HF ratio indicates that the sympathetic response is predominant, and this occurs when individuals engage in fight-or-flight behaviors or the parasympathetic response is suppressed (60). Although both males and females with depressive symptoms show the biological fight-or-flight response pattern (e.g., greater anger perception and elevated heart rate), the subsequent behavior of males and females is often different (61).

Low resilience was also strongly associated with depression; this may be a precursor to depression and is more common in females than in males (62). This study indicated that women in the MDS group showed the lowest resilience level of all. Therefore, resilience may be used to screen female students for mild depression. Additionally, increased neuroticism and decreased extraversion are characteristics of some forms of psychopathology. For example, MDD involves a combination of high neuroticism and low extraversion (63). In this study, female students with MDS showed decreased extraversion. Subjects who report decreased extraversion may typically use maladaptive strategies to regulate their emotions (e.g., avoidance, suppression, and worry) (64). These personality characteristics may play a role in the development of MDD, possibly through aberrant emotional processing (63). One systematic review found a negative relationship between measures of social rank and symptoms of depression (65). This might be partially explained by understanding the psychosocial characteristics of female students with MDS.

### Interpretation of the Correlation Analyses

The correlation analyses revealed sex-specific differences in the relationships between psychological characteristics and social cognition measures. Female students showed a positive association between the perception of negative facial stimuli (e.g., anger) and the resilience, self-esteem, and quality of life scales, whereas male students did not. Social engagement is particularly noticeable during anger regulation (66). Taken together, the present results indicate that affective issues should be considered when treating female students with MDS. Additionally, there were positive relationships between ERT UHRA and the OTS MLC4 in both males and females, which indicates that executive function decreases as the perception of anger increases in both sexes.

### Application of the ESEM Technique

The ESEM approach allows for the establishment of sex-specific predictive markers in young adults with symptoms of depression. In both male and female students, latent depression and anxiety variables were significantly predicted by PC1 (i.e., questionnaires about psychological characteristics and sleep quality). Specifically, higher scores for resilience, self-esteem, and quality of life and lower scores for hopelessness, neuroticism, and sleep quality negatively predicted the levels of depression and anxiety.

Furthermore, the Chi-squared difference tests of fit loadings, fit intercepts, and fit means according to sex revealed significant differences in the fit means. However, this result was insufficient to draw any particular conclusions or show any specific future directions. On the other hand, the difference in factor covariance between males and females might provide meaningful data because males showed significant relationships among the PC1, PC2, and PC3 factors, whereas females did not. These findings imply that it is possible to predict the degree of depression in males based on questionnaires and neurocognitive test results, whereas this prediction in females will require additional consideration (e.g., clinician interview). However, the neurocognitive test included in the present model only measures SWM; thus, it may be necessary to reevaluate the model and include other tests that measure additional neurocognitive functions.

Furthermore, the HRV indices were not included in the postulated model. Future studies should investigate the latent relationships among HRV measures, questionnaires, and neurocognitive indexes.

### Limitations

The present study has some limitations that should be considered. First, the study sample size was relatively small, and the ESEM is generally considered a large-sample analysis technique. However, the related literature includes numerous recommendations concerning the standard rule for sample size and further suggests that this rule varies, is ambiguous, and often lacks validity, suggesting that generic rules or even guidelines about appropriate sample size are extremely tricky (67). The present ESEM results represent a theoretical approach for investigating sex-specific patterns of symptoms in young adults. Therefore, further epidemiological research with broader samples will be necessary to confirm these findings. Second, the cut-off values of the STAI-S, which measures anxiety, were applied differently to males and females. Thus, it was not possible to exclude the possibility that this could have affected the characteristics of the enrolled MDS/MDD groups differentially according to sex. Third, a variety of confounding factors, including smoking status, alcohol intake, physical activity, and body mass index, that might have affected the HRV were not controlled. According to reviews of the methodologies used in HRV analyses, issues with signal analytic requirements are often under-reported despite their importance (68), and insufficient attention is paid to the environment in which data are collected (69). The present results will be more reliable after detailed control of confounding variables that may influence the interpretation of the results are instituted. Finally, all participants in the present study were university students, so the present findings may not be generalizable to young adults in the general population.

## Conclusion

The present results may be used to improve the screening of young adults with MDS before symptoms become severe and to inform prevention strategies and coordinate early treatment programs.

## Data Availability Statement

The raw data supporting the conclusions of this article will be made available by the authors, without undue reservation, to any qualified researcher.

## Ethics Statement

The studies involving human participants were reviewed and approved by the Institutional Review Board of Seoul National University College of Medicine and Hospital. The patients/participants provided their written informed consent to participate in this study.

## Author Contributions

J-AL and JJ designed the study and wrote the protocol. J-AL, JJ, J-YY, YK, HL, YC, and S-HC recruited subjects and collected clinical, neurocognitive, and physiological information. J-AL undertook data analyses. J-AL and JJ wrote the manuscript. All authors reviewed and approved the final manuscript.

## Funding

The study was supported by a grant from the Brain Research Program through the National Research Foundation of Korea (NRF) funded by the Ministry of Science (NRF-2016M3C7A1914449).

## Conflict of Interest

The authors declare that the research was conducted in the absence of any commercial or financial relationships that could be construed as a potential conflict of interest.

## Abbreviations

AIC, Akaike information criterion; ANOVA, analysis of variance; ASCII, American Standard Code for Information Interchange; BHS, Beck Hopelessness Scale; BIC, Bayesian information criterion; BIS, Barratt Impulsiveness Scale; CANTAB, Cambridge Neuropsychological Test Automated Battery; CES-D, Center for Epidemiologic Studies Depression Scale; CFI, comparative fit index; CON, control; df, degrees of freedom; ERTUHRA, Emotion Recognition Task Unbiased Hit Rate Anger; ERTUHRD, Emotion Recognition Task Unbiased Hit Rate Disgust; ERTUHRF, Emotion Recognition Task Unbiased Hit Rate Fear; ERTUHRH, Emotion Recognition Task Unbiased Hit Rate Happiness; ERTUHRS, Emotion Recognition Task Unbiased Hit Rate Sadness; ERTUHRSU, Emotion Recognition Task Unbiased Hit Rate Surprise; ESEM, exploratory structural equation modeling; FFT, Fast Fourier Transform; GAD-7, Generalized Anxiety Disorder-7; HF, high frequency; HR, heart rate; HRV, heart rate variability; LF, low frequency; M, mean; MDD, major depressive disorder; MDS, mild depressive symptoms; NEO, neuroticism-extraversion-openness; NS, not significant; OTSMCC4, One Touch Stockings of Cambridge Mean Choices to Correct (4 move); OTSMLC4, One Touch Stockings of Cambridge Mean Latency to Correct (4 move); PC, principal component; PCA, principal component analysis; PHQ-9, Patient Health Questionnaire-9; PPG, photoplethysmography; PSQI, Pittsburgh Sleep Quality Index; RAS, Resilience Appraisal Scale; RMSEA, root mean-square error of approximation; RMSSD, root mean square of successive RR interval differences; RR, time interval between successive electrocardiogram R-waves; RSES, Rosenberg Self Esteem Scale; RVPA, Rapid Visual information Processing A prime; RVPTM, Rapid Visual information Processing Total Misses; SD, standard deviation; SE, standard error; SRMR, standard root mean square residual; SS, sum of squares; SSTSSRT, Stop Signal Task Stop Signal Reaction Time; STAI-S, State-Trait Anxiety Inventory-State anxiety; SWMBE, Spatial Working Memory Between Errors; SWMS, Spatial Working Memory Strategy; SWMTE, Spatial Working Memory Total Errors; TLI, Tucker-Lewis index; WHOQOL, World Health Organization Quality of Life abbreviated version.
