# Supplementary material for: Sex-Specific Differences in Severity of Depressive Symptoms, Heart Rate Variability, and Neurocognitive Profiles of Depressed Young Adults: Exploring Characteristics for Mild Depression
Source: Front Psychiatry. 2020 Mar 17;11:217. doi: 10.3389/fpsyt.2020.00217 (PMC7092630; doi:10.3389/fpsyt.2020.00217)
Supplement: Supplementary file 1 [file Image_1.pdf]

Supplementary Material

Supplementary Figure

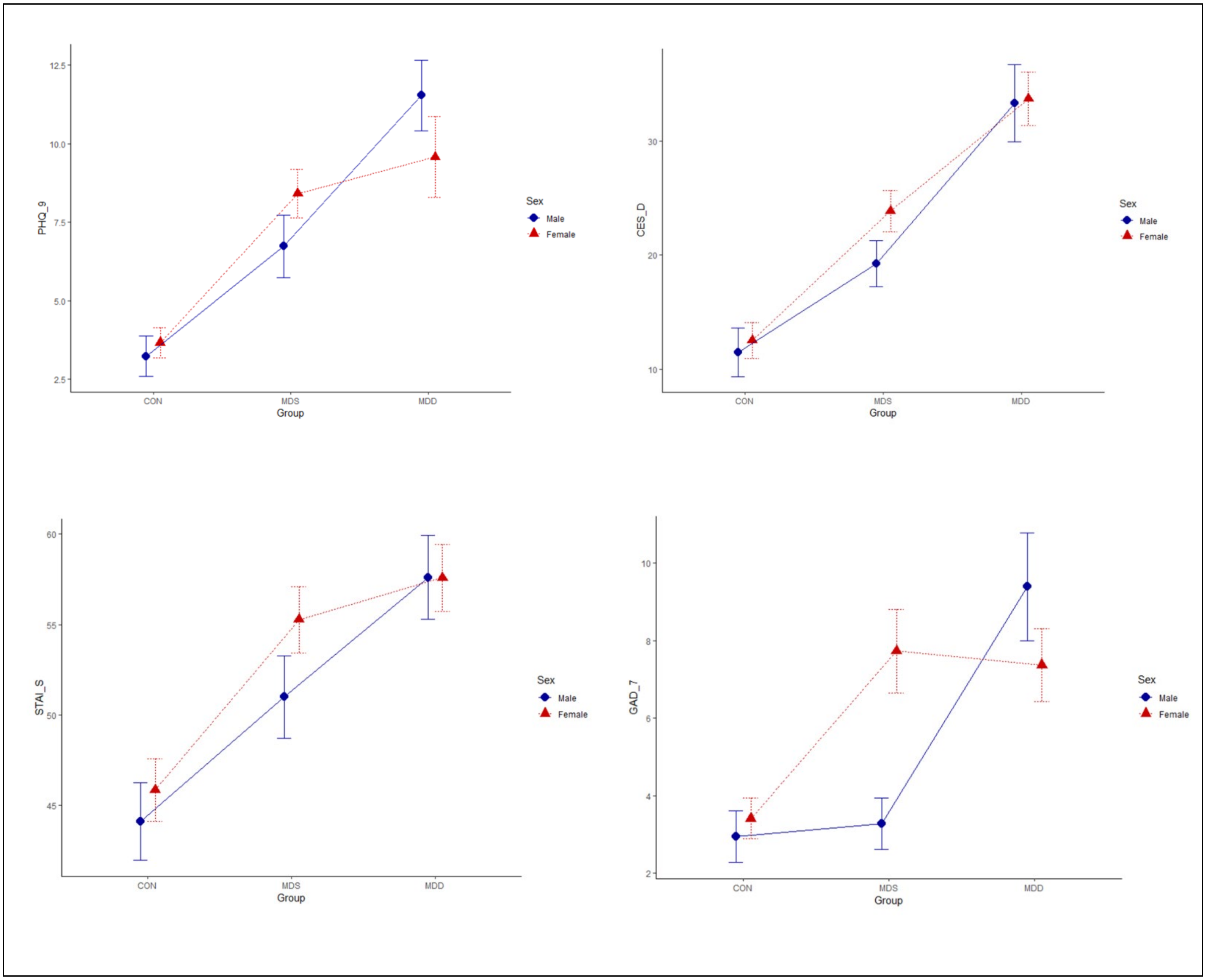

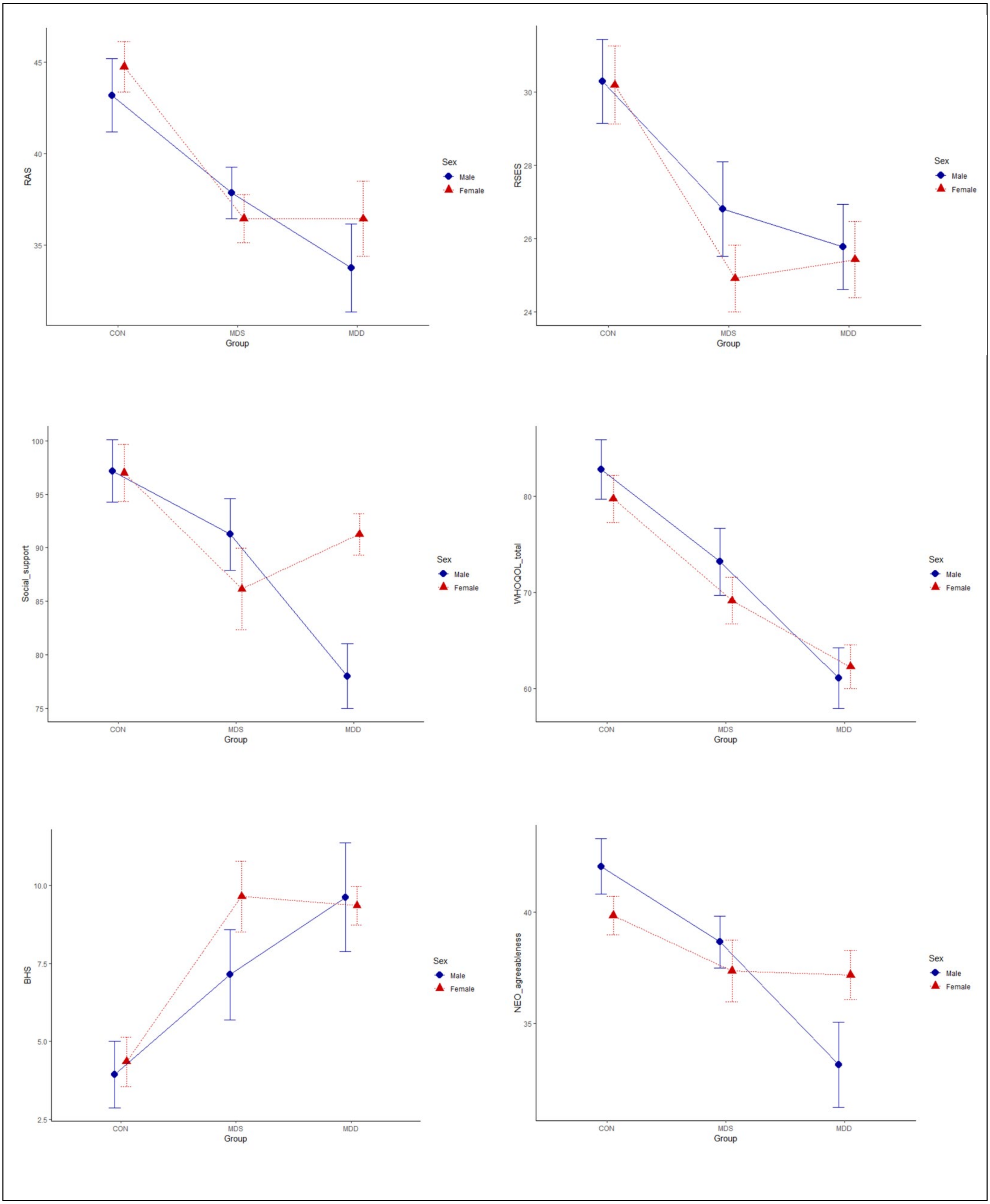
RAS
45
40
35
CON
MDS
MDD
Group
Sex
Male
Female
RSES
30
28
26
24
CON
MDS
MDD
Group
Sex
Male
Female
Social_support
100
95
90
85
80
75
CON
MDS
MDD
Group
Sex
Male
Female
WHOQOL_total
80
70
60
CON
MDS
MDD
Group
Sex
Male
Female
BHS
10.0
7.5
5.0
2.5
CON
MDS
MDD
Group
Sex
Male
Female
NEO_agreeableness
40
35
CON
MDS
MDD
Group
Sex
Male
Female

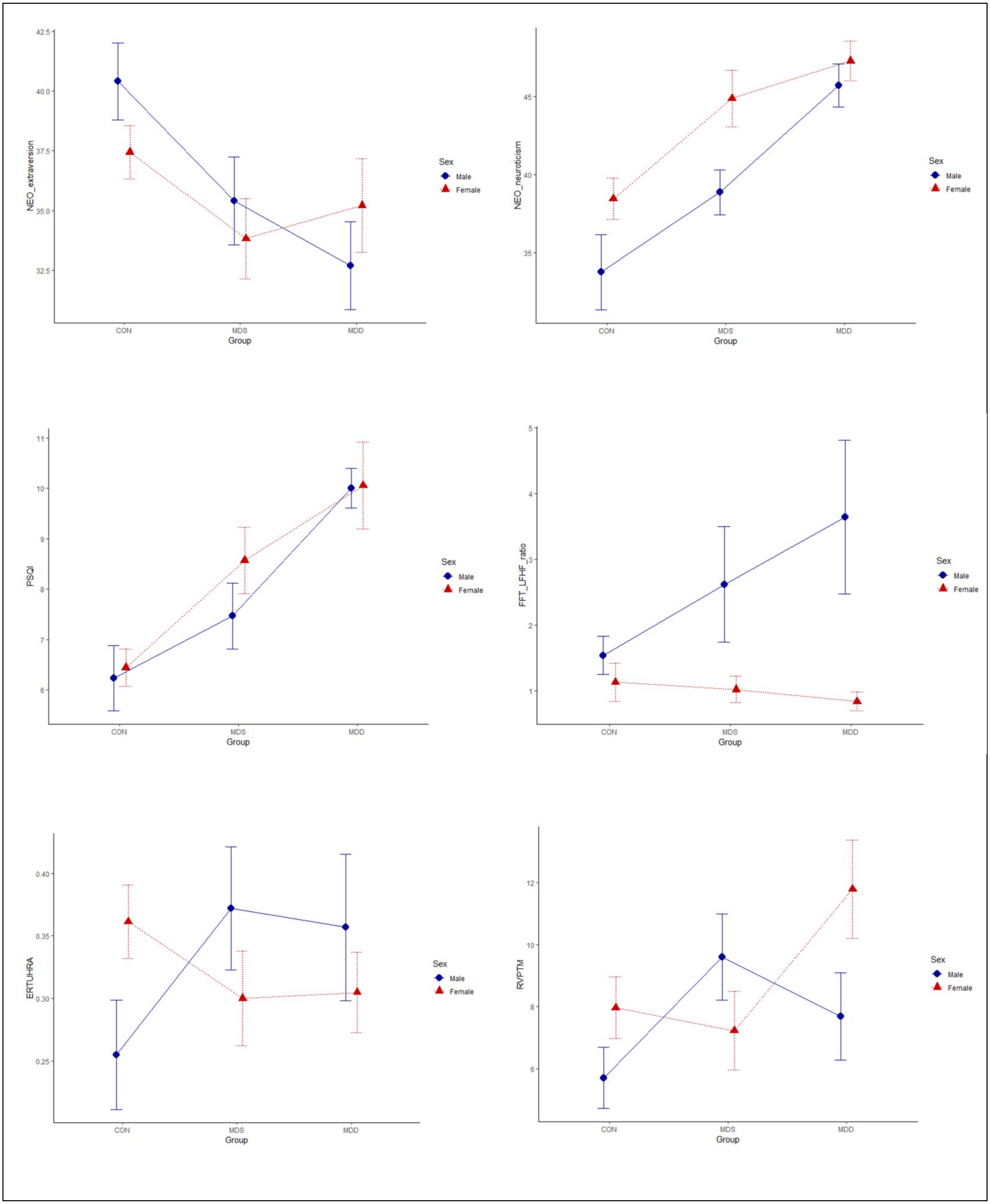

NEO_extraversion
42.5
40.0
37.5
35.0
32.5
CON
MDS
MDD
Group
Sex
Male
Female
NEO_neuroticism
45
40
35
CON
MDS
MDD
Group
Sex
Male
Female
PSQI
11
10
9
8
7
6
CON
MDS
MDD
Group
Sex
Male
Female
FFT_LFHF_ratio
5
4
3
2
1
CON
MDS
MDD
Group
Sex
Male
Female
ERTUHRA
0.40
0.35
0.30
0.25
CON
MDS
MDD
Group
Sex
Male
Female
RVPTM
12
10
8
6
CON
MDS
MDD
Group
Sex
Male
Female

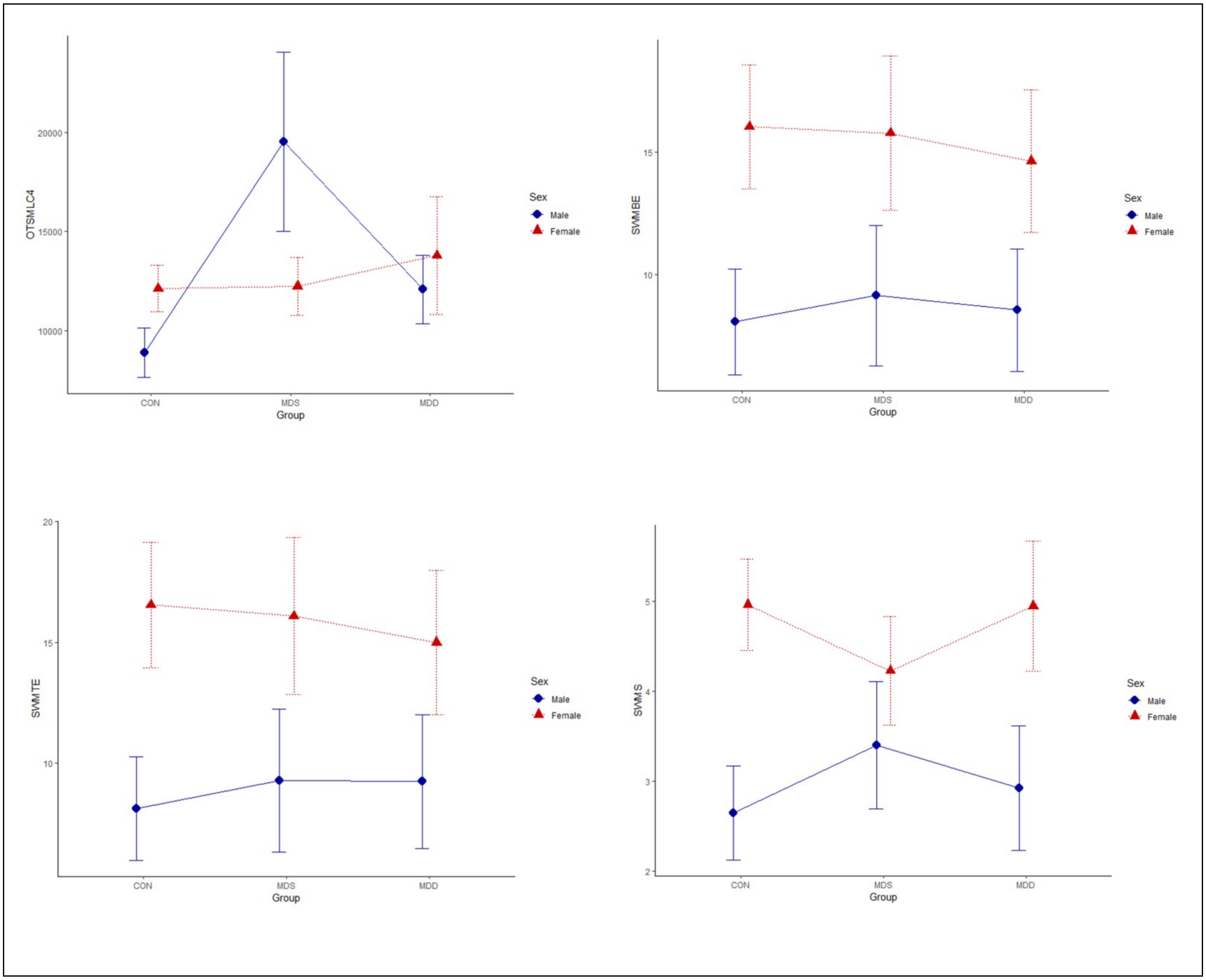

Supplementary Figure 1. Significant effects of self-reported clinical scales, HRV, and neurocognitive assessments. Results from Group x Sex interaction plot, where error bars indicate a 95% confidence interval.

HRV = Heart Rate Variability; CON = Control; MDS = Mild Depressive Symptoms; MDD = Major Depressive Disorder; PHQ-9 = Patient Health Questionnaire-9; CES-D = Center for Epidemiologic Studies Depression Scale; STAI-S = State-Trait Anxiety Inventory-State anxiety; GAD-7 = Generalized Anxiety Disorder-7; RAS = Resilience Appraisal Scale; RSES = Rosenberg Self Esteem Scale; WHOQOL = World Health Organization Quality of Life abbreviated version; BHS = Beck Hopelessness Scale; NEO = Neuroticism-Extraversion-Openness; PSQI = Pittsburgh Sleep Quality Index; FFT = Fast Fourier Transform; LF = Low Frequency; HF = High Frequency; ERTUHRA = Emotion Recognition Task Unbiased Hit Rate Anger; RVPTM = Rapid Visual information Processing Total Misses; OTSMLC4 = One Touch Stockings of Cambridge Mean Latency to Correct (4 move); SWMBE = Spatial Working Memory Between Errors; SWMTE = Spatial Working Memory Total Errors; SWMS = Spatial Working Memory Strategy.

* $P < .05$; ** $P < .01$; *** $P < .001$.
