# Supplementary material for: Sex-Specific Differences in Severity of Depressive Symptoms, Heart Rate Variability, and Neurocognitive Profiles of Depressed Young Adults: Exploring Characteristics for Mild Depression
Source: Front Psychiatry. 2020 Mar 17;11:217. doi: 10.3389/fpsyt.2020.00217 (PMC7092630; doi:10.3389/fpsyt.2020.00217)
Supplement: Supplementary file 2 [file Table_1.docx]

Supplementary Material

# Supplementary Table 1. Correlation between clinical characteristic and neurocognitive and heart rate variability

|  | **Male** |  |  |  |
| --- | --- | --- | --- | --- |
| **#** | **row** | **column** | ***r*** | ***P*** |
| 1 | ZFFT_LFHF_ratio | ZERTUHRA | -0.057 | 0.732 |
| 2 | ZFFT_LFHF_ratio | ZOTSMLC4 | -0.071 | 0.669 |
| 3 | ZERTUHRA | ZOTSMLC4 | 0.348 | 0.030 |
| 4 | ZFFT_LFHF_ratio | ZSWMBE | -0.079 | 0.634 |
| 5 | ZERTUHRA | ZSWMBE | -0.159 | 0.333 |
| 6 | ZOTSMLC4 | ZSWMBE | 0.161 | 0.327 |
| 7 | ZFFT_LFHF_ratio | ZSWMTE | -0.069 | 0.676 |
| 8 | ZERTUHRA | ZSWMTE | -0.179 | 0.277 |
| 9 | ZOTSMLC4 | ZSWMTE | 0.145 | 0.378 |
| 10 | ZSWMBE | ZSWMTE | 0.995 | 0.000 |
| 11 | ZFFT_LFHF_ratio | ZSWMS | -0.125 | 0.448 |
| 12 | ZERTUHRA | ZSWMS | -0.027 | 0.869 |
| 13 | ZOTSMLC4 | ZSWMS | 0.148 | 0.368 |
| 14 | ZSWMBE | ZSWMS | 0.746 | 0.000 |
| 15 | ZSWMTE | ZSWMS | 0.727 | 0.000 |
| 16 | ZFFT_LFHF_ratio | ZCES_D | 0.097 | 0.558 |
| 17 | ZERTUHRA | ZCES_D | 0.112 | 0.498 |
| 18 | ZOTSMLC4 | ZCES_D | 0.120 | 0.467 |
| 19 | ZSWMBE | ZCES_D | -0.128 | 0.436 |
| 20 | ZSWMTE | ZCES_D | -0.107 | 0.518 |
| 21 | ZSWMS | ZCES_D | -0.157 | 0.339 |
| 22 | ZFFT_LFHF_ratio | ZPHQ_9 | 0.055 | 0.739 |
| 23 | ZERTUHRA | ZPHQ_9 | 0.030 | 0.854 |
| 24 | ZOTSMLC4 | ZPHQ_9 | 0.230 | 0.159 |
| 25 | ZSWMBE | ZPHQ_9 | -0.032 | 0.847 |
| 26 | ZSWMTE | ZPHQ_9 | -0.011 | 0.946 |
| 27 | ZSWMS | ZPHQ_9 | -0.051 | 0.756 |
| 28 | ZCES_D | ZPHQ_9 | 0.713 | 0.000 |
| 29 | ZFFT_LFHF_ratio | ZGAD_7 | 0.033 | 0.840 |
| 30 | ZERTUHRA | ZGAD_7 | -0.106 | 0.522 |
| 31 | ZOTSMLC4 | ZGAD_7 | 0.160 | 0.331 |
| 32 | ZSWMBE | ZGAD_7 | 0.102 | 0.538 |
| 33 | ZSWMTE | ZGAD_7 | 0.117 | 0.480 |
| 34 | ZSWMS | ZGAD_7 | 0.068 | 0.682 |
| 35 | ZCES_D | ZGAD_7 | 0.585 | 0.000 |
| 36 | ZPHQ_9 | ZGAD_7 | 0.791 | 0.000 |
| 37 | ZFFT_LFHF_ratio | ZSTAI_S | 0.090 | 0.586 |
| 38 | ZERTUHRA | ZSTAI_S | 0.098 | 0.553 |
| 39 | ZOTSMLC4 | ZSTAI_S | 0.334 | 0.038 |
| 40 | ZSWMBE | ZSTAI_S | -0.064 | 0.697 |
| 41 | ZSWMTE | ZSTAI_S | -0.055 | 0.741 |
| 42 | ZSWMS | ZSTAI_S | -0.048 | 0.773 |
| 43 | ZCES_D | ZSTAI_S | 0.660 | 0.000 |
| 44 | ZPHQ_9 | ZSTAI_S | 0.802 | 0.000 |
| 45 | ZGAD_7 | ZSTAI_S | 0.768 | 0.000 |
| 46 | ZFFT_LFHF_ratio | ZRAS | -0.027 | 0.871 |
| 47 | ZERTUHRA | ZRAS | -0.104 | 0.528 |
| 48 | ZOTSMLC4 | ZRAS | -0.111 | 0.501 |
| 49 | ZSWMBE | ZRAS | 0.201 | 0.221 |
| 50 | ZSWMTE | ZRAS | 0.176 | 0.283 |
| 51 | ZSWMS | ZRAS | 0.145 | 0.377 |
| 52 | ZCES_D | ZRAS | -0.679 | 0.000 |
| 53 | ZPHQ_9 | ZRAS | -0.492 | 0.001 |
| 54 | ZGAD_7 | ZRAS | -0.468 | 0.003 |
| 55 | ZSTAI_S | ZRAS | -0.612 | 0.000 |
| 56 | ZFFT_LFHF_ratio | ZRSES | 0.057 | 0.731 |
| 57 | ZERTUHRA | ZRSES | 0.048 | 0.770 |
| 58 | ZOTSMLC4 | ZRSES | -0.360 | 0.024 |
| 59 | ZSWMBE | ZRSES | 0.037 | 0.825 |
| 60 | ZSWMTE | ZRSES | 0.027 | 0.869 |
| 61 | ZSWMS | ZRSES | 0.102 | 0.535 |
| 62 | ZCES_D | ZRSES | -0.554 | 0.000 |
| 63 | ZPHQ_9 | ZRSES | -0.635 | 0.000 |
| 64 | ZGAD_7 | ZRSES | -0.462 | 0.003 |
| 65 | ZSTAI_S | ZRSES | -0.635 | 0.000 |
| 66 | ZRAS | ZRSES | 0.564 | 0.000 |
| 67 | ZFFT_LFHF_ratio | ZSocial_support | -0.143 | 0.385 |
| 68 | ZERTUHRA | ZSocial_support | -0.245 | 0.132 |
| 69 | ZOTSMLC4 | ZSocial_support | 0.013 | 0.936 |
| 70 | ZSWMBE | ZSocial_support | 0.384 | 0.016 |
| 71 | ZSWMTE | ZSocial_support | 0.345 | 0.032 |
| 72 | ZSWMS | ZSocial_support | 0.177 | 0.281 |
| 73 | ZCES_D | ZSocial_support | -0.639 | 0.000 |
| 74 | ZPHQ_9 | ZSocial_support | -0.348 | 0.030 |
| 75 | ZGAD_7 | ZSocial_support | -0.278 | 0.087 |
| 76 | ZSTAI_S | ZSocial_support | -0.313 | 0.052 |
| 77 | ZRAS | ZSocial_support | 0.508 | 0.001 |
| 78 | ZRSES | ZSocial_support | 0.201 | 0.220 |
| 79 | ZFFT_LFHF_ratio | ZWHOQOL_total | -0.032 | 0.848 |
| 80 | ZERTUHRA | ZWHOQOL_total | -0.098 | 0.554 |
| 81 | ZOTSMLC4 | ZWHOQOL_total | 0.000 | 0.998 |
| 82 | ZSWMBE | ZWHOQOL_total | 0.246 | 0.132 |
| 83 | ZSWMTE | ZWHOQOL_total | 0.216 | 0.187 |
| 84 | ZSWMS | ZWHOQOL_total | 0.201 | 0.219 |
| 85 | ZCES_D | ZWHOQOL_total | -0.792 | 0.000 |
| 86 | ZPHQ_9 | ZWHOQOL_total | -0.687 | 0.000 |
| 87 | ZGAD_7 | ZWHOQOL_total | -0.516 | 0.001 |
| 88 | ZSTAI_S | ZWHOQOL_total | -0.629 | 0.000 |
| 89 | ZRAS | ZWHOQOL_total | 0.498 | 0.001 |
| 90 | ZRSES | ZWHOQOL_total | 0.486 | 0.002 |
| 91 | ZSocial_support | ZWHOQOL_total | 0.711 | 0.000 |
| 92 | ZFFT_LFHF_ratio | ZBHS | -0.065 | 0.693 |
| 93 | ZERTUHRA | ZBHS | 0.195 | 0.235 |
| 94 | ZOTSMLC4 | ZBHS | 0.121 | 0.462 |
| 95 | ZSWMBE | ZBHS | -0.264 | 0.105 |
| 96 | ZSWMTE | ZBHS | -0.239 | 0.143 |
| 97 | ZSWMS | ZBHS | -0.228 | 0.162 |
| 98 | ZCES_D | ZBHS | 0.625 | 0.000 |
| 99 | ZPHQ_9 | ZBHS | 0.469 | 0.003 |
| 100 | ZGAD_7 | ZBHS | 0.202 | 0.217 |
| 101 | ZSTAI_S | ZBHS | 0.408 | 0.010 |
| 102 | ZRAS | ZBHS | -0.460 | 0.003 |
| 103 | ZRSES | ZBHS | -0.651 | 0.000 |
| 104 | ZSocial_support | ZBHS | -0.578 | 0.000 |
| 105 | ZWHOQOL_total | ZBHS | -0.624 | 0.000 |
| 106 | ZFFT_LFHF_ratio | ZNEO_agreeableness | -0.166 | 0.312 |
| 107 | ZERTUHRA | ZNEO_agreeableness | -0.208 | 0.204 |
| 108 | ZOTSMLC4 | ZNEO_agreeableness | -0.084 | 0.612 |
| 109 | ZSWMBE | ZNEO_agreeableness | 0.135 | 0.412 |
| 110 | ZSWMTE | ZNEO_agreeableness | 0.100 | 0.543 |
| 111 | ZSWMS | ZNEO_agreeableness | 0.076 | 0.644 |
| 112 | ZCES_D | ZNEO_agreeableness | -0.528 | 0.001 |
| 113 | ZPHQ_9 | ZNEO_agreeableness | -0.415 | 0.009 |
| 114 | ZGAD_7 | ZNEO_agreeableness | -0.377 | 0.018 |
| 115 | ZSTAI_S | ZNEO_agreeableness | -0.392 | 0.014 |
| 116 | ZRAS | ZNEO_agreeableness | 0.467 | 0.003 |
| 117 | ZRSES | ZNEO_agreeableness | 0.282 | 0.082 |
| 118 | ZSocial_support | ZNEO_agreeableness | 0.654 | 0.000 |
| 119 | ZWHOQOL_total | ZNEO_agreeableness | 0.421 | 0.008 |
| 120 | ZBHS | ZNEO_agreeableness | -0.414 | 0.009 |
| 121 | ZFFT_LFHF_ratio | ZNEO_extraversion | 0.226 | 0.167 |
| 122 | ZERTUHRA | ZNEO_extraversion | -0.282 | 0.082 |
| 123 | ZOTSMLC4 | ZNEO_extraversion | -0.028 | 0.866 |
| 124 | ZSWMBE | ZNEO_extraversion | 0.327 | 0.042 |
| 125 | ZSWMTE | ZNEO_extraversion | 0.298 | 0.065 |
| 126 | ZSWMS | ZNEO_extraversion | 0.191 | 0.243 |
| 127 | ZCES_D | ZNEO_extraversion | -0.380 | 0.017 |
| 128 | ZPHQ_9 | ZNEO_extraversion | -0.200 | 0.223 |
| 129 | ZGAD_7 | ZNEO_extraversion | -0.008 | 0.961 |
| 130 | ZSTAI_S | ZNEO_extraversion | -0.100 | 0.545 |
| 131 | ZRAS | ZNEO_extraversion | 0.310 | 0.055 |
| 132 | ZRSES | ZNEO_extraversion | 0.189 | 0.249 |
| 133 | ZSocial_support | ZNEO_extraversion | 0.691 | 0.000 |
| 134 | ZWHOQOL_total | ZNEO_extraversion | 0.545 | 0.000 |
| 135 | ZBHS | ZNEO_extraversion | -0.580 | 0.000 |
| 136 | ZNEO_agreeableness | ZNEO_extraversion | 0.374 | 0.019 |
| 137 | ZFFT_LFHF_ratio | ZNEO_neuroticism | 0.137 | 0.404 |
| 138 | ZERTUHRA | ZNEO_neuroticism | -0.118 | 0.473 |
| 139 | ZOTSMLC4 | ZNEO_neuroticism | 0.150 | 0.361 |
| 140 | ZSWMBE | ZNEO_neuroticism | -0.168 | 0.306 |
| 141 | ZSWMTE | ZNEO_neuroticism | -0.152 | 0.355 |
| 142 | ZSWMS | ZNEO_neuroticism | -0.262 | 0.108 |
| 143 | ZCES_D | ZNEO_neuroticism | 0.758 | 0.000 |
| 144 | ZPHQ_9 | ZNEO_neuroticism | 0.687 | 0.000 |
| 145 | ZGAD_7 | ZNEO_neuroticism | 0.640 | 0.000 |
| 146 | ZSTAI_S | ZNEO_neuroticism | 0.684 | 0.000 |
| 147 | ZRAS | ZNEO_neuroticism | -0.586 | 0.000 |
| 148 | ZRSES | ZNEO_neuroticism | -0.685 | 0.000 |
| 149 | ZSocial_support | ZNEO_neuroticism | -0.385 | 0.015 |
| 150 | ZWHOQOL_total | ZNEO_neuroticism | -0.556 | 0.000 |
| 151 | ZBHS | ZNEO_neuroticism | 0.507 | 0.001 |
| 152 | ZNEO_agreeableness | ZNEO_neuroticism | -0.436 | 0.005 |
| 153 | ZNEO_extraversion | ZNEO_neuroticism | -0.158 | 0.336 |
| 154 | ZFFT_LFHF_ratio | ZPSQI | 0.001 | 0.993 |
| 155 | ZERTUHRA | ZPSQI | -0.034 | 0.838 |
| 156 | ZOTSMLC4 | ZPSQI | 0.167 | 0.310 |
| 157 | ZSWMBE | ZPSQI | -0.038 | 0.818 |
| 158 | ZSWMTE | ZPSQI | -0.029 | 0.859 |
| 159 | ZSWMS | ZPSQI | -0.133 | 0.418 |
| 160 | ZCES_D | ZPSQI | 0.461 | 0.003 |
| 161 | ZPHQ_9 | ZPSQI | 0.699 | 0.000 |
| 162 | ZGAD_7 | ZPSQI | 0.578 | 0.000 |
| 163 | ZSTAI_S | ZPSQI | 0.576 | 0.000 |
| 164 | ZRAS | ZPSQI | -0.201 | 0.220 |
| 165 | ZRSES | ZPSQI | -0.451 | 0.004 |
| 166 | ZSocial_support | ZPSQI | -0.189 | 0.249 |
| 167 | ZWHOQOL_total | ZPSQI | -0.500 | 0.001 |
| 168 | ZBHS | ZPSQI | 0.186 | 0.256 |
| 169 | ZNEO_agreeableness | ZPSQI | -0.382 | 0.016 |
| 170 | ZNEO_extraversion | ZPSQI | -0.188 | 0.251 |
| 171 | ZNEO_neuroticism | ZPSQI | 0.527 | 0.001 |
|  | **Female** |  |  |  |
| **#** | **row** | **column** | ***r*** | ***P*** |
| 1 | ZFFT_LFHF_ratio | ZERTUHRA | -0.049 | 0.716 |
| 2 | ZFFT_LFHF_ratio | ZOTSMLC4 | 0.014 | 0.918 |
| 3 | ZERTUHRA | ZOTSMLC4 | 0.338 | 0.010 |
| 4 | ZFFT_LFHF_ratio | ZSWMBE | 0.071 | 0.598 |
| 5 | ZERTUHRA | ZSWMBE | 0.253 | 0.058 |
| 6 | ZOTSMLC4 | ZSWMBE | 0.232 | 0.083 |
| 7 | ZFFT_LFHF_ratio | ZSWMTE | 0.066 | 0.625 |
| 8 | ZERTUHRA | ZSWMTE | 0.235 | 0.078 |
| 9 | ZOTSMLC4 | ZSWMTE | 0.237 | 0.076 |
| 10 | ZSWMBE | ZSWMTE | 0.998 | 0.000 |
| 11 | ZFFT_LFHF_ratio | ZSWMS | 0.033 | 0.809 |
| 12 | ZERTUHRA | ZSWMS | 0.004 | 0.977 |
| 13 | ZOTSMLC4 | ZSWMS | 0.017 | 0.899 |
| 14 | ZSWMBE | ZSWMS | 0.720 | 0.000 |
| 15 | ZSWMTE | ZSWMS | 0.721 | 0.000 |
| 16 | ZFFT_LFHF_ratio | ZCES_D | -0.081 | 0.551 |
| 17 | ZERTUHRA | ZCES_D | -0.253 | 0.057 |
| 18 | ZOTSMLC4 | ZCES_D | -0.051 | 0.708 |
| 19 | ZSWMBE | ZCES_D | -0.130 | 0.334 |
| 20 | ZSWMTE | ZCES_D | -0.129 | 0.338 |
| 21 | ZSWMS | ZCES_D | -0.004 | 0.977 |
| 22 | ZFFT_LFHF_ratio | ZPHQ_9 | -0.080 | 0.556 |
| 23 | ZERTUHRA | ZPHQ_9 | -0.171 | 0.202 |
| 24 | ZOTSMLC4 | ZPHQ_9 | -0.152 | 0.259 |
| 25 | ZSWMBE | ZPHQ_9 | -0.186 | 0.167 |
| 26 | ZSWMTE | ZPHQ_9 | -0.173 | 0.199 |
| 27 | ZSWMS | ZPHQ_9 | -0.174 | 0.196 |
| 28 | ZCES_D | ZPHQ_9 | 0.539 | 0.000 |
| 29 | ZFFT_LFHF_ratio | ZGAD_7 | -0.145 | 0.283 |
| 30 | ZERTUHRA | ZGAD_7 | -0.156 | 0.248 |
| 31 | ZOTSMLC4 | ZGAD_7 | -0.175 | 0.193 |
| 32 | ZSWMBE | ZGAD_7 | -0.360 | 0.006 |
| 33 | ZSWMTE | ZGAD_7 | -0.360 | 0.006 |
| 34 | ZSWMS | ZGAD_7 | -0.337 | 0.010 |
| 35 | ZCES_D | ZGAD_7 | 0.437 | 0.001 |
| 36 | ZPHQ_9 | ZGAD_7 | 0.715 | 0.000 |
| 37 | ZFFT_LFHF_ratio | ZSTAI_S | -0.146 | 0.277 |
| 38 | ZERTUHRA | ZSTAI_S | -0.261 | 0.050 |
| 39 | ZOTSMLC4 | ZSTAI_S | -0.037 | 0.782 |
| 40 | ZSWMBE | ZSTAI_S | -0.152 | 0.258 |
| 41 | ZSWMTE | ZSTAI_S | -0.151 | 0.263 |
| 42 | ZSWMS | ZSTAI_S | -0.114 | 0.397 |
| 43 | ZCES_D | ZSTAI_S | 0.677 | 0.000 |
| 44 | ZPHQ_9 | ZSTAI_S | 0.685 | 0.000 |
| 45 | ZGAD_7 | ZSTAI_S | 0.768 | 0.000 |
| 46 | ZFFT_LFHF_ratio | ZRAS | -0.066 | 0.624 |
| 47 | ZERTUHRA | ZRAS | 0.335 | 0.011 |
| 48 | ZOTSMLC4 | ZRAS | 0.108 | 0.423 |
| 49 | ZSWMBE | ZRAS | 0.028 | 0.836 |
| 50 | ZSWMTE | ZRAS | 0.015 | 0.914 |
| 51 | ZSWMS | ZRAS | 0.076 | 0.573 |
| 52 | ZCES_D | ZRAS | -0.455 | 0.000 |
| 53 | ZPHQ_9 | ZRAS | -0.463 | 0.000 |
| 54 | ZGAD_7 | ZRAS | -0.404 | 0.002 |
| 55 | ZSTAI_S | ZRAS | -0.557 | 0.000 |
| 56 | ZFFT_LFHF_ratio | ZRSES | 0.022 | 0.871 |
| 57 | ZERTUHRA | ZRSES | 0.336 | 0.011 |
| 58 | ZOTSMLC4 | ZRSES | 0.247 | 0.064 |
| 59 | ZSWMBE | ZRSES | 0.096 | 0.477 |
| 60 | ZSWMTE | ZRSES | 0.097 | 0.473 |
| 61 | ZSWMS | ZRSES | -0.056 | 0.680 |
| 62 | ZCES_D | ZRSES | -0.631 | 0.000 |
| 63 | ZPHQ_9 | ZRSES | -0.424 | 0.001 |
| 64 | ZGAD_7 | ZRSES | -0.465 | 0.000 |
| 65 | ZSTAI_S | ZRSES | -0.665 | 0.000 |
| 66 | ZRAS | ZRSES | 0.661 | 0.000 |
| 67 | ZFFT_LFHF_ratio | ZSocial_support | -0.038 | 0.777 |
| 68 | ZERTUHRA | ZSocial_support | 0.178 | 0.184 |
| 69 | ZOTSMLC4 | ZSocial_support | 0.042 | 0.757 |
| 70 | ZSWMBE | ZSocial_support | 0.113 | 0.401 |
| 71 | ZSWMTE | ZSocial_support | 0.114 | 0.400 |
| 72 | ZSWMS | ZSocial_support | 0.159 | 0.237 |
| 73 | ZCES_D | ZSocial_support | -0.152 | 0.260 |
| 74 | ZPHQ_9 | ZSocial_support | -0.229 | 0.087 |
| 75 | ZGAD_7 | ZSocial_support | -0.358 | 0.006 |
| 76 | ZSTAI_S | ZSocial_support | -0.421 | 0.001 |
| 77 | ZRAS | ZSocial_support | 0.421 | 0.001 |
| 78 | ZRSES | ZSocial_support | 0.288 | 0.030 |
| 79 | ZFFT_LFHF_ratio | ZWHOQOL_total | 0.122 | 0.367 |
| 80 | ZERTUHRA | ZWHOQOL_total | 0.267 | 0.045 |
| 81 | ZOTSMLC4 | ZWHOQOL_total | 0.061 | 0.650 |
| 82 | ZSWMBE | ZWHOQOL_total | 0.183 | 0.172 |
| 83 | ZSWMTE | ZWHOQOL_total | 0.183 | 0.172 |
| 84 | ZSWMS | ZWHOQOL_total | 0.075 | 0.581 |
| 85 | ZCES_D | ZWHOQOL_total | -0.652 | 0.000 |
| 86 | ZPHQ_9 | ZWHOQOL_total | -0.585 | 0.000 |
| 87 | ZGAD_7 | ZWHOQOL_total | -0.536 | 0.000 |
| 88 | ZSTAI_S | ZWHOQOL_total | -0.712 | 0.000 |
| 89 | ZRAS | ZWHOQOL_total | 0.608 | 0.000 |
| 90 | ZRSES | ZWHOQOL_total | 0.640 | 0.000 |
| 91 | ZSocial_support | ZWHOQOL_total | 0.491 | 0.000 |
| 92 | ZFFT_LFHF_ratio | ZBHS | -0.088 | 0.514 |
| 93 | ZERTUHRA | ZBHS | -0.249 | 0.062 |
| 94 | ZOTSMLC4 | ZBHS | -0.108 | 0.423 |
| 95 | ZSWMBE | ZBHS | -0.068 | 0.613 |
| 96 | ZSWMTE | ZBHS | -0.073 | 0.590 |
| 97 | ZSWMS | ZBHS | -0.187 | 0.163 |
| 98 | ZCES_D | ZBHS | 0.530 | 0.000 |
| 99 | ZPHQ_9 | ZBHS | 0.347 | 0.008 |
| 100 | ZGAD_7 | ZBHS | 0.375 | 0.004 |
| 101 | ZSTAI_S | ZBHS | 0.522 | 0.000 |
| 102 | ZRAS | ZBHS | -0.441 | 0.001 |
| 103 | ZRSES | ZBHS | -0.597 | 0.000 |
| 104 | ZSocial_support | ZBHS | -0.255 | 0.055 |
| 105 | ZWHOQOL_total | ZBHS | -0.481 | 0.000 |
| 106 | ZFFT_LFHF_ratio | ZNEO_agreeableness | 0.019 | 0.886 |
| 107 | ZERTUHRA | ZNEO_agreeableness | -0.095 | 0.483 |
| 108 | ZOTSMLC4 | ZNEO_agreeableness | 0.010 | 0.944 |
| 109 | ZSWMBE | ZNEO_agreeableness | -0.029 | 0.828 |
| 110 | ZSWMTE | ZNEO_agreeableness | -0.025 | 0.856 |
| 111 | ZSWMS | ZNEO_agreeableness | -0.011 | 0.933 |
| 112 | ZCES_D | ZNEO_agreeableness | -0.317 | 0.016 |
| 113 | ZPHQ_9 | ZNEO_agreeableness | -0.128 | 0.344 |
| 114 | ZGAD_7 | ZNEO_agreeableness | -0.207 | 0.122 |
| 115 | ZSTAI_S | ZNEO_agreeableness | -0.224 | 0.093 |
| 116 | ZRAS | ZNEO_agreeableness | 0.325 | 0.014 |
| 117 | ZRSES | ZNEO_agreeableness | 0.202 | 0.131 |
| 118 | ZSocial_support | ZNEO_agreeableness | 0.322 | 0.015 |
| 119 | ZWHOQOL_total | ZNEO_agreeableness | 0.372 | 0.004 |
| 120 | ZBHS | ZNEO_agreeableness | -0.053 | 0.693 |
| 121 | ZFFT_LFHF_ratio | ZNEO_extraversion | -0.091 | 0.502 |
| 122 | ZERTUHRA | ZNEO_extraversion | 0.110 | 0.414 |
| 123 | ZOTSMLC4 | ZNEO_extraversion | 0.213 | 0.112 |
| 124 | ZSWMBE | ZNEO_extraversion | 0.149 | 0.268 |
| 125 | ZSWMTE | ZNEO_extraversion | 0.168 | 0.212 |
| 126 | ZSWMS | ZNEO_extraversion | 0.021 | 0.877 |
| 127 | ZCES_D | ZNEO_extraversion | -0.270 | 0.042 |
| 128 | ZPHQ_9 | ZNEO_extraversion | -0.157 | 0.243 |
| 129 | ZGAD_7 | ZNEO_extraversion | -0.154 | 0.254 |
| 130 | ZSTAI_S | ZNEO_extraversion | -0.278 | 0.036 |
| 131 | ZRAS | ZNEO_extraversion | 0.227 | 0.089 |
| 132 | ZRSES | ZNEO_extraversion | 0.345 | 0.009 |
| 133 | ZSocial_support | ZNEO_extraversion | 0.188 | 0.161 |
| 134 | ZWHOQOL_total | ZNEO_extraversion | 0.308 | 0.020 |
| 135 | ZBHS | ZNEO_extraversion | -0.426 | 0.001 |
| 136 | ZNEO_agreeableness | ZNEO_extraversion | 0.218 | 0.103 |
| 137 | ZFFT_LFHF_ratio | ZNEO_neuroticism | -0.055 | 0.682 |
| 138 | ZERTUHRA | ZNEO_neuroticism | -0.171 | 0.204 |
| 139 | ZOTSMLC4 | ZNEO_neuroticism | -0.044 | 0.742 |
| 140 | ZSWMBE | ZNEO_neuroticism | -0.176 | 0.189 |
| 141 | ZSWMTE | ZNEO_neuroticism | -0.172 | 0.200 |
| 142 | ZSWMS | ZNEO_neuroticism | -0.110 | 0.415 |
| 143 | ZCES_D | ZNEO_neuroticism | 0.592 | 0.000 |
| 144 | ZPHQ_9 | ZNEO_neuroticism | 0.515 | 0.000 |
| 145 | ZGAD_7 | ZNEO_neuroticism | 0.627 | 0.000 |
| 146 | ZSTAI_S | ZNEO_neuroticism | 0.730 | 0.000 |
| 147 | ZRAS | ZNEO_neuroticism | -0.488 | 0.000 |
| 148 | ZRSES | ZNEO_neuroticism | -0.625 | 0.000 |
| 149 | ZSocial_support | ZNEO_neuroticism | -0.333 | 0.011 |
| 150 | ZWHOQOL_total | ZNEO_neuroticism | -0.640 | 0.000 |
| 151 | ZBHS | ZNEO_neuroticism | 0.466 | 0.000 |
| 152 | ZNEO_agreeableness | ZNEO_neuroticism | -0.424 | 0.001 |
| 153 | ZNEO_extraversion | ZNEO_neuroticism | -0.164 | 0.223 |
| 154 | ZFFT_LFHF_ratio | ZPSQI | -0.080 | 0.554 |
| 155 | ZERTUHRA | ZPSQI | 0.036 | 0.791 |
| 156 | ZOTSMLC4 | ZPSQI | 0.035 | 0.797 |
| 157 | ZSWMBE | ZPSQI | 0.012 | 0.931 |
| 158 | ZSWMTE | ZPSQI | 0.030 | 0.826 |
| 159 | ZSWMS | ZPSQI | -0.013 | 0.924 |
| 160 | ZCES_D | ZPSQI | 0.329 | 0.012 |
| 161 | ZPHQ_9 | ZPSQI | 0.494 | 0.000 |
| 162 | ZGAD_7 | ZPSQI | 0.264 | 0.047 |
| 163 | ZSTAI_S | ZPSQI | 0.291 | 0.028 |
| 164 | ZRAS | ZPSQI | -0.336 | 0.011 |
| 165 | ZRSES | ZPSQI | -0.177 | 0.189 |
| 166 | ZSocial_support | ZPSQI | -0.110 | 0.416 |
| 167 | ZWHOQOL_total | ZPSQI | -0.333 | 0.011 |
| 168 | ZBHS | ZPSQI | 0.316 | 0.017 |
| 169 | ZNEO_agreeableness | ZPSQI | -0.214 | 0.110 |
| 170 | ZNEO_extraversion | ZPSQI | -0.124 | 0.360 |
| 171 | ZNEO_neuroticism | ZPSQI | 0.167 | 0.214 |

FFT = Fast Fourier Transform; LF = Low Frequency; HF = High Frequency; ERTUHRA = Emotion Recognition Task Unbiased Hit Rate Anger; OTSMLC4 = One Touch Stockings of Cambridge Mean Latency to Correct (4 move); SWMBE = Spatial Working Memory Between Errors; SWMTE = Spatial Working Memory Total Errors; SWMS = Spatial Working Memory Strategy; PHQ-9 = Patient Health Questionnaire-9; CES-D = Center for Epidemiologic Studies Depression Scale; STAI-S = State-Trait Anxiety Inventory-State anxiety; GAD-7 = Generalized Anxiety Disorder-7; RAS = Resilience Appraisal Scale; RSES = Rosenberg Self Esteem Scale; WHOQOL = World Health Organization Quality of Life abbreviated version; BHS = Beck Hopelessness Scale; NEO = Neuroticism-Extraversion-Openness; PSQI = Pittsburgh Sleep Quality Index.
