# Supplementary material for: Sex-Specific Differences in Severity of Depressive Symptoms, Heart Rate Variability, and Neurocognitive Profiles of Depressed Young Adults: Exploring Characteristics for Mild Depression
Source: Front Psychiatry. 2020 Mar 17;11:217. doi: 10.3389/fpsyt.2020.00217 (PMC7092630; doi:10.3389/fpsyt.2020.00217)
Supplement: Supplementary file 3 [file Table_2.docx]

Supplementary Material

# Supplementary Table 2. Rotated factor loadings for principal component analysis

| **Variable** | **PC1** | **PC2** | **PC3** |
| --- | --- | --- | --- |
| ZFFT_LFHF_ratio | -0.02 | -0.19 | 0.00 |
| ZERTUHRA | -0.29 | 0.14 | -0.60 |
| ZOTSMLC4 | -0.02 | 0.29 | -0.29 |
| ZSWMBE | -0.09 | **0.96** | 0.04 |
| ZSWMTE | -0.08 | **0.96** | 0.04 |
| ZSWMS | -0.05 | **0.84** | 0.08 |
| ZCES_D | **0.79** | -0.01 | -0.23 |
| ZPHQ_9 | **0.82** | -0.06 | 0.03 |
| ZGAD_7 | **0.79** | -0.12 | 0.11 |
| ZSTAI_S | **0.88** | -0.01 | -0.05 |
| ZRAS | **-0.68** | 0.06 | 0.21 |
| ZRSES | **-0.78** | -0.02 | 0.06 |
| ZSocial_support | -0.39 | 0.24 | **0.56** |
| ZWHOQOL_total | **-0.78** | 0.12 | 0.31 |
| ZBHS | **0.58** | -0.10 | -0.40 |
| ZNEO_agreeableness | -0.36 | -0.03 | **0.58** |
| ZNEO_extraversion | -0.24 | 0.16 | **0.61** |
| ZNEO_neuroticism | **0.81** | -0.04 | -0.07 |
| ZPSQI | **0.52** | 0.12 | -0.09 |

Rotated factor loading ≥ 0.50 considered to indicate significant; Significant variable is remarked as a bold.

PC = Principal Component; FFT = Fast Fourier Transform; LF = Low Frequency; HF = High Frequency; ERTUHRA = Emotion Recognition Task Unbiased Hit Rate Anger; OTSMLC4 = One Touch Stockings of Cambridge Mean Latency to Correct (4 move); SWMBE = Spatial Working Memory Between Errors; SWMTE = Spatial Working Memory Total Errors; SWMS = Spatial Working Memory Strategy; CES-D = Center for Epidemiologic Studies Depression Scale; PHQ-9 = Patient Health Questionnaire-9; GAD-7 = Generalized Anxiety Disorder-7; STAI-S = State-Trait Anxiety Inventory-State anxiety; RAS = Resilience Appraisal Scale; RSES = Rosenberg Self Esteem Scale; WHOQOL = World Health Organization Quality of Life abbreviated version; BHS = Beck Hopelessness Scale; NEO = Neuroticism-Extraversion-Openness; PSQI = Pittsburgh Sleep Quality Index.
